# Neurotransmitter-stimulated neuron-derived sEVs have opposite effects on amyloid β-induced neuronal damage

**DOI:** 10.1186/s12951-021-01070-5

**Published:** 2021-10-15

**Authors:** Yunxiao Dou, Junchao Xie, Yan Tan, Min Zhang, Yanxin Zhao, Xueyuan Liu

**Affiliations:** Department of Neurology, Shanghai Tenth People’s Hospital, Tongji University School of Medicine, 301 Middle Yanchang Road, Shanghai, 200072 China

**Keywords:** Excitatory/inhibitory imbalance, Neurotransmitters, Alzheimer’s disease, Amyloid β, Gamma-aminobutyric acid, Glutamate, Extracellular vesicles (EVs)

## Abstract

The ratio of excitatory to inhibitory neurotransmitters is essential for maintaining the firing patterns of neural networks, and is strictly regulated within individual neurons and brain regions. Excitatory to inhibitory (E/I) imbalance has been shown to participate in the progression of neurodegenerative diseases, including Alzheimer's disease (AD). Glutamate excitotoxicity and GABAergic neuron dysfunction appear to be key components of the neuronal cell death that takes place in AD. Since extracellular vesicles (EVs) are now explored as an important vehicle in transmitting signals between cells, we hypothesized that the function of neuron-derived small EVs (sEVs) might be regulated by the status of neurotransmitter balance and that sEVs might affect amyloid β (Aβ) toxicity on neurons. This study aimed to reveal the effects of sEVs from unbalanced neurotransmitter-stimulated neurons on Aβ-induced toxicity. We demonstrated the opposite effects of the two groups of sEVs isolated from neurons stimulated by glutamate or GABA on Aβ toxicity in vivo and in vitro. The sEVs released from GABA-treated neurons alleviated Aβ-induced damage, while those released from glutamate-treated neurons aggravated Aβ toxicity. Furthermore, we compared the microRNA (miRNA) composition of sEVs isolated from glutamate/GABA/PBS-treated neurons. Our results showed that glutamate and GABA oppositely regulated miR-132 levels in sEVs, resulting in the opposite destiny of recipient cells challenged with Aβ. Our results indicated that manipulating the function of sEVs by different neurotransmitters may reveal the mechanisms underlying the pathogenesis of AD and provide a promising strategy for AD treatment.

**Supplementary Information:**

The online version contains supplementary material available at 10.1186/s12951-021-01070-5.

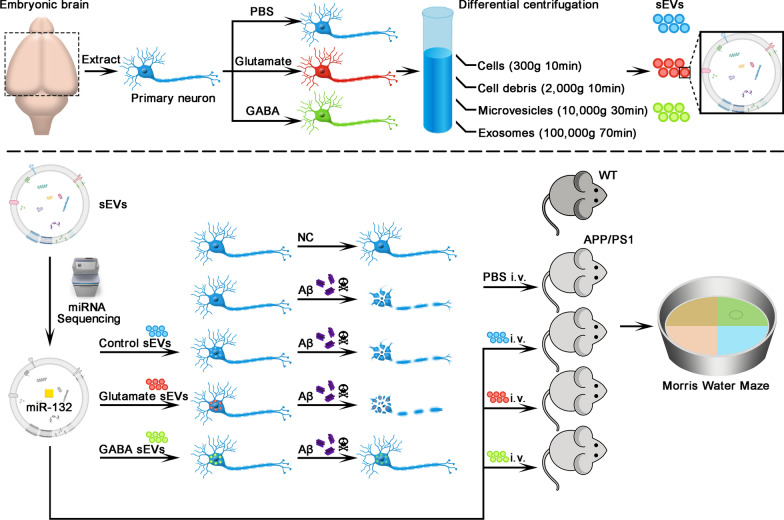

## Introduction

When neurons communicate, they release excitatory and inhibitory neurotransmitters to activate or inhibit other neurons that share synapses [1–3]. The excitatory to inhibitory (E/I) balance fine-tunes neural network activity in a narrow time window by adjusting the intensity and weight of E/I neurotransmission related to external stimuli [4]. Currently, the E/I imbalance hypothesis is conceptualized as a disequilibrium between glutamatergic and GABAergic synaptic inputs. E/I imbalance has been postulated to underlie brain dysfunction across neurodegenerative diseases, including Alzheimer’s disease (AD), Parkinson’s disease (PD), schizophrenia, and other nervous diseases [5, 6]. Glutamate excitotoxicity and GABAergic neuron dysfunction appear to be key components of the neuronal cell death that takes place in AD. There are remaining questions to be answered concerning how E/I imbalance contributes to the pathology of AD. Since neuron-derived extracellular vesicles (EVs) are now explored as an important vehicle in transmitting signals between neurons, we hypothesized that the function of EVs might be regulated by the status of neurotransmitter balance and that EVs might affect Aβ toxicity in neurons.

EVs are natural in origin and thus less immunogenic and well tolerated in body fluids [7]. EVs are heterogeneous cell-derived membranous vesicles that are commonly grouped into three types, small EVs (sEVs) (50–150 nm), large EVs (100–1000 nm) and apoptotic bodies (up to 5 μm) based on their size and mode of biogenesis [8]. These sEVs can breach the blood-brain barrier (BBB), emerge as key mediators of the communication among central nervous system (CNS) cell types [9]. sEVs are applied in conventional and therapeutic research and allow cell–cell transfer of proteins or RNAs [10, 11]. Evidence has indicated that microRNAs (miRNAs) in sEVs contribute to many cellular and biological processes, such as neuronal cell growth and apoptosis, thus affecting different functional processes, such as learning and memory [12]. Interestingly, sEVs have also been proven to allow trans-synaptic communication [13] and mediate the spreading of functional molecules [13]. Thus, in this study, primary cultured neurons were treated with glutamate/GABA/PBS, and sEVs were isolated. Next, sEVs from different sources were added to neurons treated with Aβ or injected into AD model mice. The destiny of mice and neurons treated with Aβ were then evaluated. The sEVs released from GABA-treated neurons alleviated Aβ-induced damage, while those released from glutamate-treated neurons aggravated Aβ toxicity. Furthermore, we compared the miRNA composition of sEVs isolated from glutamate/GABA/PBS-treated neurons via miRNA sequencing. The study further indicated that the changes in miR-132 in sEVs contribute to the biochemical alterations that characterize the pathology. Our results suggest that engineering sEVs biologically by manipulating the GABAergic system may be a useful strategy to prevent or alleviate the pathogenesis of AD.

## Results

### Glutamate and GABA stimulate neurons to release sEVs

**Fig. 1:**
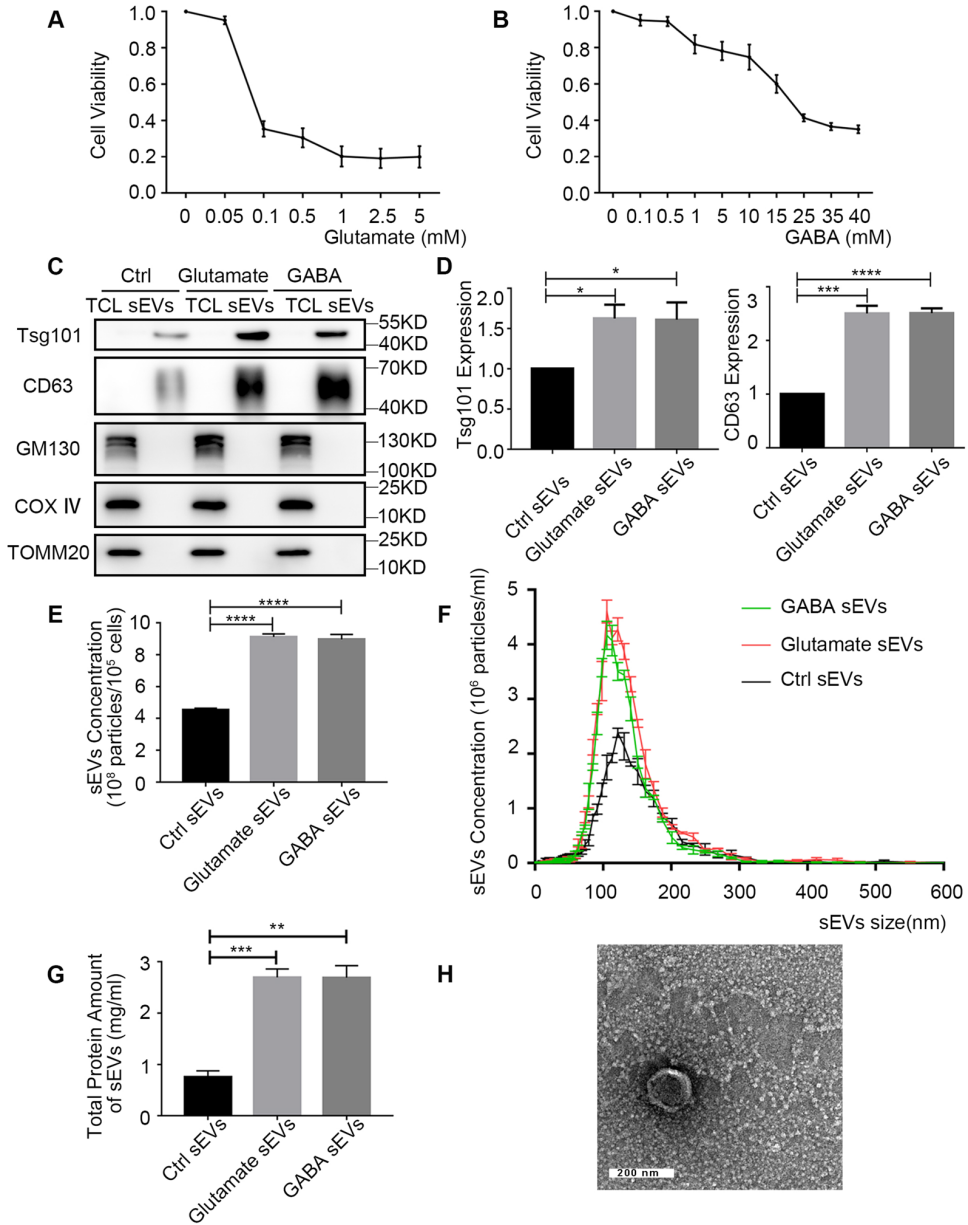
Characterization of sEVs released from glutamate or GABA-treated neurons. **A**, **B** Primary cultured neurons on DIV 15 were treated with a gradient concentration of glutamate (**A**) or GABA (**B**) and subjected to a CCK-8 cell viability assay. **C**–**H** Neurons were cultured in a T75 culture flask. The number of cells and the volume of culture medium in the three groups were consistent. After neurons on DIV 15 were treated with 10 μM glutamate (diluted in 20 μL PBS), 300 μM GABA (diluted in 20 μL PBS), or PBS (20 μL) for 24 h, sEVs were extracted by serial ultracentrifugation from cell culture supernatants from equivalent numbers of cells. **C** The positive transmembrane sEV marker CD63, the cytosolic sEV marker Tsg101, and the negative controls GM130, COX IV and TOMM20 were detected in the sEVs and their derived cells by Western blot. sEVs: Neuron-derived sEVs, TCL: Total cell lysates. **D** Quantification and statistics of CD63 and Tsg101 protein levels detected by Western blot from three independent experiments. **E** Quantification and statistics of the concentration of the nanoparticles detected by nanoparticle tracking analysis (NTA) from three independent experiments. **F** Representative NTA traces of sEVs were derived from three experimental groups. **G** The total protein amount of the sEVs in each group were detected by the BCA method. **H** The morphology of sEVs from PBS-treated neurons was observed under a transmission electron microscope. n = 3. Data are presented as the mean ± SEM, *p < 0.05, **p < 0.01, ***p < 0.001, ****p < 0.0001

We investigated whether glutamate and GABA trigger neuronal sEV release. The dissociated neurons contain glutamatergic and GABAergic neurons, forming a functional network within the second week of culture [14]. Thus, primary cultured neurons on Day 15 in vitro (DIV15) were treated with a gradient concentration of glutamate or GABA for 24 h to verify their effects on cell viability. Additional file 1: Fig. S1A shows the primary cortical neurons on DIV15. We found that the viability of neurons decreased with increasing concentrations of both neurotransmitters (Fig. 1A, B). Specifically, cell viability began to decline when the concentrations of glutamate and GABA were > 0.02 mM and 1 mM, respectively. In the following experiments, primary cultured neurons on DIV 15 were treated with 10 μM glutamate or 300 μM GABA. The sEVs were isolated from the same number of neurons in each group. Additionally, sEVs isolated from the supernatant of an equal volume of PBS-treated neurons served as control sEVs (Ctrl sEVs). The positive transmembrane sEV marker CD63 and the cytosolic sEV marker Tsg101 were utilized to identify sEVs by Western blot. Moreover, GM130, COX IV and TOMM20 were used as the negative controls in Fig. 1C to ascertain the absence of contaminating intracellular material in sEV preps. These results showed that the amounts of CD63 and Tsg101 detected in the sEVs of glutamate- or GABA-treated cells were significantly increased compared to those in the sEVs of PBS-treated cells (Fig. 1D). Consistently, nanoparticle tracking analysis (NTA) of the pellets obtained by ultracentrifugation showed an increase in the number of particles secreted by glutamate/GABA-treated cells compared to that in control cells, indicating that a larger number of sEVs were secreted (Fig. 1E, F). In addition, the bicinchoninic acid (BCA) method was used to detect the total protein amount of the sEVs in each group (Fig. 1G). The results also suggest that GABA and glutamate promote the release of sEVs by neurons, which was consistent with the NTA results.

Furthermore, NTA (Fig. 1F) and transmission electron microscopic (Fig. 1H; Additional file 1: Fig. S1C) analyses revealed that the size of the majority of vesicles is similar to those generally described for sEVs (i.e., 50–150 nm).

### sEVs derived from GABA or glutamate-treated neurons alleviate or aggravate Aβ-induced damage, respectively

**Fig. 2:**
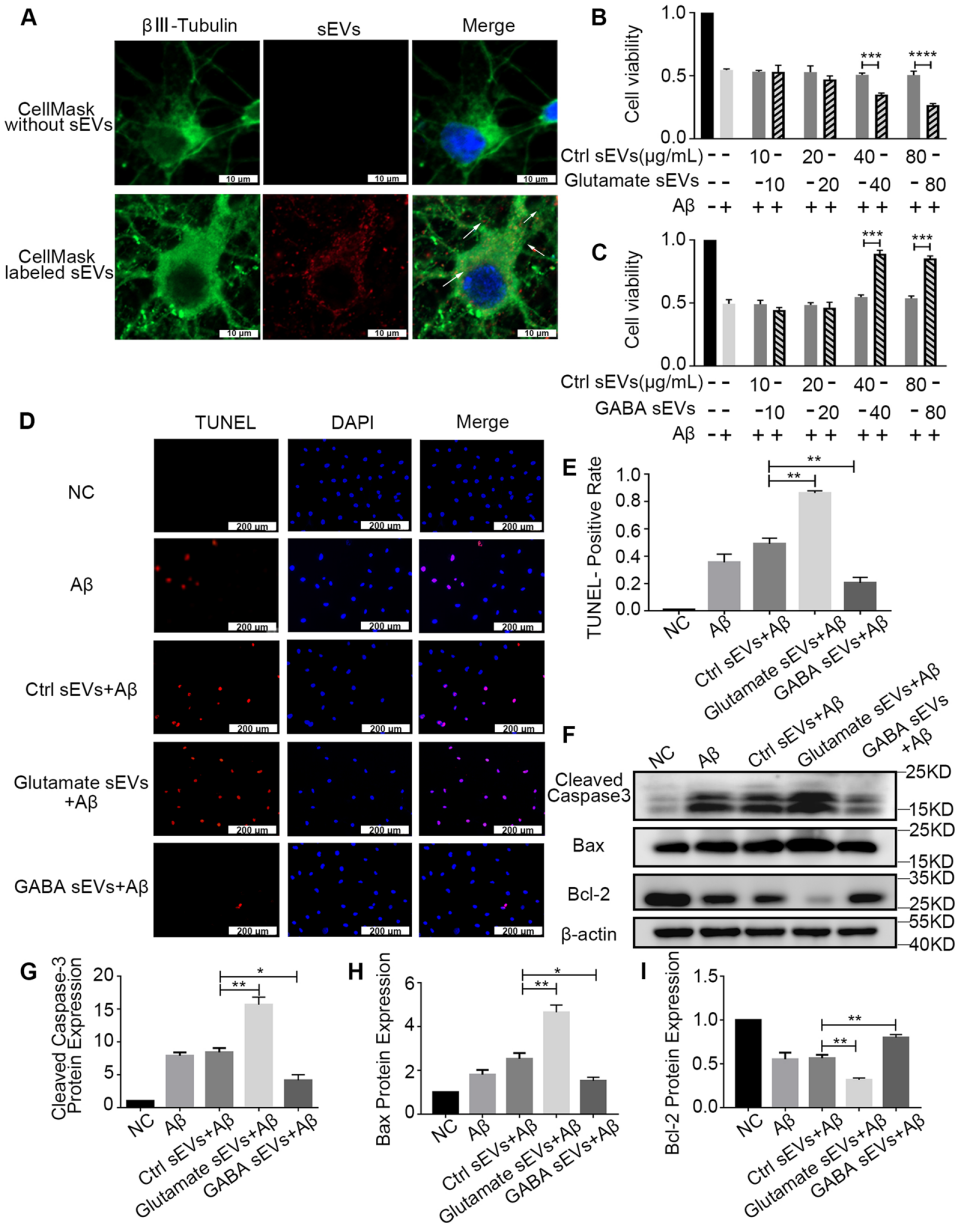
Neuron-internalized sEVs alleviate or aggravate Aβ damage to neurons. **A** sEVs were labeled with CellMask (red) and added to the primary neurons. A control group (only CellMask without sEVs) was established to exclude false-positive staining caused by the dye. Then, the neurons were immunostained with βIII-tubulin (green) and visualized under a confocal microscope. CellMask-labeled sEVs were indicated by white arrows. **B**, **C** After the pretreatment of primary neurons with the indicated concentration (μg/mL) of glutamate sEVs, GABA sEVs, or Ctrl sEVs for 24 h, the neurons were treated with 0.5 μM Aβ_1–42_ for 48 h. Cell viability was measured using CCK-8. **D**, **E** Cell apoptosis levels were measured using TUNEL assay. **F**–**I** The expression of apoptotic molecules (cleaved Caspase-3, Bax, and Bcl-2) was detected by Western blot. The relative protein expression was normalized to that of β-actin quantified by ImageJ software. n = 3. Data are presented as the mean ± SEM, *p < 0.05, **p < 0.01, ***p < 0.001, ****p < 0.0001

We further analyzed whether released sEVs can be internalized by other neurons. The neurons were cultured and treated with glutamate (10 μM) or GABA (300 μM), while PBS was used as a control. Then, glutamate sEVs, GABA sEVs, and Ctrl sEVs were isolated and labeled with the fluorescence dye CellMask or PKH26 [15]. Additionally, a control group (only dye without sEVs) was established to exclude the false-positive staining. The uptake of sEVs was visualized after 24 h. The internalization of red fluorescent sEVs was visible at the somatodendritic and axonal domains of the neurons in the CellMask-labeled sEV-treated groups (Fig. 2A) and PKH26-labeled sEV-treated groups (Additional file 2: Fig. S2A), whereas the control remained dark. Thus, the incubation of neurons with sEVs led to an uptake of these particles.

To investigate whether sEV delivery could change the effect of Aβ on neurons, we detected the viability of cultured neurons exposed to sEVs for 24 h and then treated them with or without Aβ. Glutamate sEVs, GABA sEVs, and Ctrl sEVs were isolated as described previously. The toxicity of Aβ was confirmed by detecting the viability of primary neurons treated with Aβ_1–42_ for 48 h. Aβ_1–42_ significantly decreased neuron viability in a dose-dependent manner with a median lethal dose of 0.5 μM (Additional file 3: Fig. S3A). Then, the primary cultured neurons were exposed to a gradient concentration of sEVs for 24 h and treated with 0.5 μM Aβ_1–42_ for another 48 h. In addition, primary neurons without any treatment comprised the negative control group. The neurons exposed to glutamate sEVs (total sEV protein concentration ≥ 40 μg/mL) and Aβ exhibited significantly lower viability than those exposed to Ctrl sEVs and Aβ (Fig. 2B), indicating that the sEVs released from glutamate-stimulated neurons exacerbate Aβ-induced neuron toxicity. Conversely, the viability of neurons stimulated with GABA sEVs (total sEV protein concentration ≥ 40 μg/mL) was significantly higher than that of neurons treated with Ctrl sEVs (Fig. 2C).

In addition, the neurons were incubated as mentioned above and subjected to TUNEL staining. The percentage of TUNEL-positive cells in the GABA sEV-treated group was significantly lower than that in the Ctrl sEV-treated group. In contrast, TUNEL-positive cells were prominently higher in the glutamate sEV group than in the Ctrl sEV group (Fig. 2D, E).

To verify that the result is predictable rather than accidental, we evaluated the effects of sEVs derived from neurons treated with a higher or lower concentration of glutamate/GABA. Neurons were treated with PBS or different concentrations of glutamate (5 μM and 15 μM) or GABA (100 μM or 500 μM). The sEVs were then isolated from each group, and their effects on Aβ-induced neuron toxicity were examined. As shown in Additional file 3: Fig. S3B–I, neurons subjected to GABA sEVs and Aβ exhibited significantly higher viability and fewer TUNEL-positive cells than those treated with Ctrl sEVs and Aβ; in contrast, neurons treated with glutamate sEVs showed significantly lower viability and more TUNEL-positive cells. These findings implied that sEVs could be internalized by other neurons and could alter the fate of Aβ-treated neurons.

Furthermore, the expression levels of apoptotic molecules (cleaved Caspase-3, Bax, and Bcl-2) were detected. The Western blot results in Fig. 2F–I demonstrated that treatment with GABA sEVs significantly downregulated the expression of cleaved Caspase-3 and Bax and upregulated Bcl-2 expression in neurons compared to Ctrl sEVs. On the other hand, the glutamate sEV treatment significantly upregulated cleaved Caspase-3 and Bax expression and suppressed Bcl-2 expression. These data supported the theory that sEVs released from neurons induced by glutamate or GABA could aggravate or alleviate Aβ damage to neurons, respectively.

### sEVs from neurons treated by different transmitters alleviate or deteriorate spatial memory deficits in APP/PS1 mice

To monitor the function of sEVs secreted by glutamate/GABA-stimulated neurons in vivo, we employed APPswe/PSEN1dE9 (APP/PS1) mice. APP/PS1 mice are double transgenic mice that express a chimeric mouse/human amyloid precursor protein (Mo/HuAPP695swe) and mutant human presenilin 1 (PS1-dE9), and both highlight neurons in the CNS. The mutations are associated with early-onset AD [16].

**Fig. 3:**
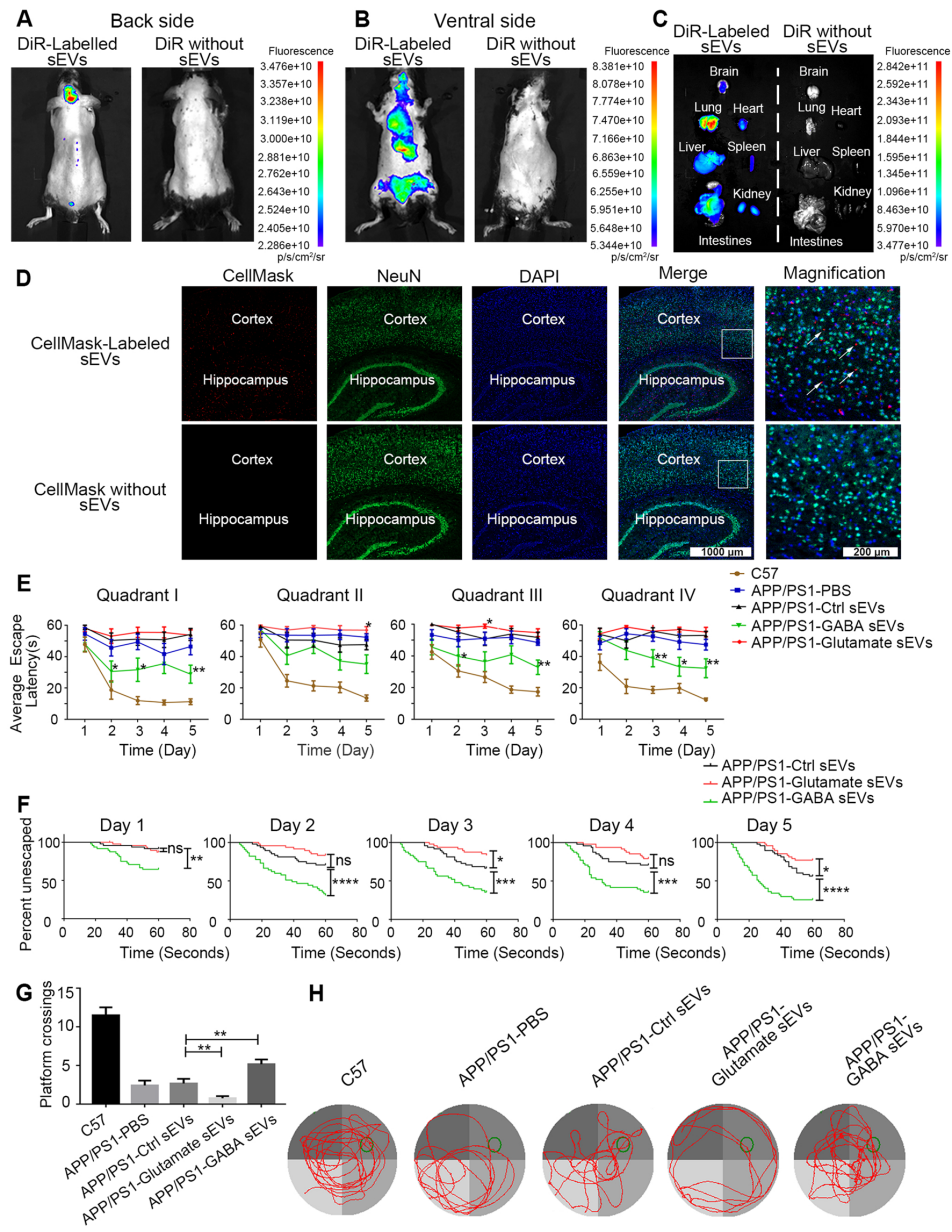
sEVs Distribution and function in spatial learning and memory function of APP/PS1 mice after injection. **A**–**C** Live mice were imaged using an in vivo imaging system 24 h after DiR-labeled sEVs were injected. A control group (only DiR without sEVs) was established to exclude the false-positive staining caused by the dye. **A**, **B** Imaging of the backside and the ventral side, respectively. **C** The indicated organs were harvested and imaged with the in vivo imaging system. **D** The sEVs were labeled with the fluorescence dye CellMask (red) and injected via the tail vein in the mice. Brain slices were produced and subjected to the immunostaining of the neuron marker NeuN (green) and visualized. The control group mice were injected with CellMask only without sEVs. N = 3. **E**–**H** APP/PS1 mice were injected with Ctrl sEVs (APP/PS1-Ctrl sEV group), glutamate sEVs (APP/PS1-Glutamate sEV group) or GABA sEVs (APP/PS1-GABA sEV group) through tail vein at the same time every other day. The dosage of sEVs was 1.0 × 10^10^ particles/g body weight (p/g). In addition, C57BL/6 wild-type (C57 group) and AD model group with an equivalent volume of PBS injection (APP/PS1-PBS group) were included as the negative and positive controls, respectively. After 40 days, spatial learning and memory were evaluated based on the MWM performance (N = 12/group). During Days 1–5, the time that elapsed until the mice reached the platform was noted. * represents p < 0.05, ** represents p < 0.01 compared with APP/PS1-Ctrl sEV Group (**E**). For latency to escape, Kaplan–Meier survival analysis with the Mantel-Cox log-rank test was employed to account for the non-normal distribution of latencies resulting from the 60-s maximum trial duration (**F**). On Day 6, the number of times that the animals swam over the platform location (**G**) and the pathways that elapsed were evaluated (**H**). The green circle represents the platform, and the red curve shows the swimming path of the mice. Data are represented as the mean ± SEM, *p < 0.05, **p < 0.01, ***p < 0.001, ****p < 0.0001

To assess the distribution of sEVs systemically delivered in mice, the near-infrared dye 1, 1′-dioctadecyl-3, 3, 3′, 3′-tetramethylindotricarbocyanine iodide (DiR) [17, 18] was used owing to its robust near-infrared light penetrating the tissues. A negative control experiment (inject only DiR without sEVs) was also performed. Subsequently, DiR-labeled sEVs were injected via the tail vein of APP/PS1 mice. Images of live mice were captured 24 h after the injection using an in vivo imaging system (Fig. 3A, B) to assess the biodistribution. Fluorescence was detected in the brain and vital organs of mice with DiR-labeled sEVs. The organs were harvested and imaged to identify the organ of the origin of the fluorescence signal and minimize signal interference. In addition, to rule out the possibility of monitoring free dye, mice were treated with free DiR without sEVs prior to organ harvest. As shown in Fig. 3C, the brain, heart, liver, lung, spleen, intestine, and kidneys showed different degrees of fluorescence. Importantly, these results suggested that we tracked labeled sEVs and not merely free dye in the tissues.

Subsequently, the brain was removed and tested to assess whether the neurons internalized the sEVs. The sEVs were labeled with CellMask or PKH26 before injection. As shown in Fig. 3D and Additional file 4: Fig. S4A, the images of brain slices provide evidence of neuron-internalizing sEVs, including in the cortex. Dye CellMask- or PKH26-labeled sEVs (red) and NeuN^+^-neurons (green) overlapped in brain slices. Mice injected with dye without sEVs were used as a negative control.

APP/PS1 mice (20 weeks old) were injected with glutamate sEVs (APP/PS1-glutamate sEVs group) or GABA sEVs (APP/PS1-GABA sEVs group) through the tail vein at the same time every other day for 40 days to monitor the function of sEVs secreted by glutamate/GABA-stimulated neurons in vivo. APP/PS1 mice were injected with Ctrl sEVs as a vehicle (APP/PS1-Ctrl sEVs group). Moreover, the normal mouse C57BL/6 wild-type (C57 group) and AD model groups treated with PBS (APP/PS1-PBS group) were included as negative and positive controls, respectively. Then, spatial learning and memory were evaluated based on the Morris water maze (MWM) performance in five groups (N = 12 in each group). The behavioral training protocol was presented in the MWM method. The time (escape latency) that elapsed until the mice reached the platform was noted during acquisition trials (Days 1–5). The number of times that the animals swam over the platform location and the pathways that elapsed were counted during the probe test (Day 6). Next, we compared the learning curves in the four quadrants of the five groups. As shown in Fig. 3E, during the learning phase, the average escape latency of the APP/PS1-GABA sEV group mice was significantly shorter than that of APP/PS1-Ctrl sEV group mice from Day 2 in quadrant I and III, while the average escape latency of the APP/PS1-Glutamate sEV group mice was significantly longer than that of the APP/PS1-Ctrl sEV group mice on Day 5 in quadrant II. Furthermore, for latency to escape, Kaplan–Meier survival analysis with the Mantel-Cox log-rank test was employed to account for the non-normal distribution of latencies resulting from the 60-s maximum trial duration according to the methods described previously [19, 20]. We concluded that the APP/PS1-glutamate sEV group mice took significantly longer to escape on Days 3 and 5 of spatial acquisition than the control mice, as determined by the log-rank test (Fig. 3F). As depicted in Fig. 3G and H, during the probe test, the APP/PS1-GABA sEV group mice crossed the platform (the green circle) more often than the APP/PS1-Ctrl sEVs group mice while the APP/PS1-Glutamate sEVs group mice crossed the platform fewer times. As shown in Fig. 3H, APP/PS1-GABA sEVs group mice spent significantly more time in the platform quadrant, while APP/PS1-Ctrl sEVs group animals and APP/PS1-Glutamate sEVs group mice swam randomly. Thus, we concluded that GABA treatment could rescue the spatial memory deficits in APP/PS1 mice while glutamate treatment of the sEVs could deteriorate it.

**Fig. 4:**
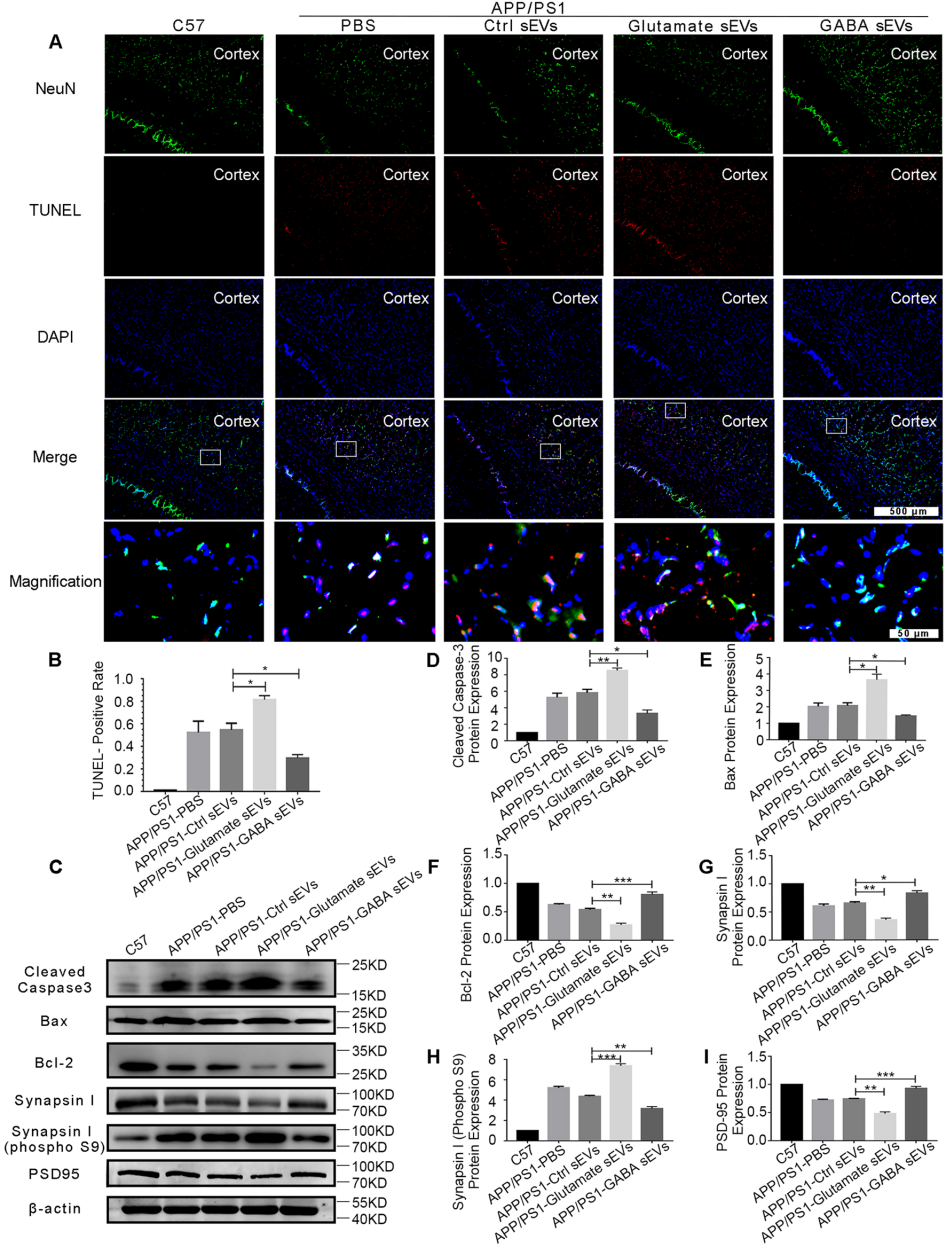
Effects of sEVs from neurons treated with different transmitters on neuron apoptosis of APP/PS1 mice. APP/PS1 mice were injected with Ctrl sEVs (APP/PS1-Ctrl sEV group), glutamate sEVs (APP/PS1-glutamate sEV group) or GABA sEVs (APP/PS1-GABA sEV group) through tail vein. C57/BL wild-type (C57 group) and AD model groups with an equivalent volume of PBS injection (APP/PS1-PBS group) were included as the negative and positive controls, respectively. The dosage was the same as above. **A**, **B** Forty days later, the proportion of TUNEL-positive cells in brain slices was detected. Red shows TUNEL-positive cells. Green indicates NeuN-positive cells, neurons. **C**–**I** The cerebral cortex was excised to examine the expression of apoptotic and synapse-related proteins. Western blot results of cleaved Caspase-3, Bax, Bcl-2, Synapsin I, Synapsin I (phospho S9), and PSD-95 in the brain tissue. N = 3. Data are represented as the mean ± SEM, *p < 0.05, **p < 0.01, ***p < 0.001

In addition, we detected the percentage of TUNEL-positive cells in brain slices. The results were consistent with the changes observed in the in vitro experiment. The percentage of positive cells in the Ctrl sEV group was lower than that in the glutamate sEV group but higher than that in the GABA sEV group (Fig. 4A, B).

Furthermore, apoptosis molecules (cleaved Caspase-3, Bax, and Bcl-2) and synapse-related proteins (Synapsin I, Synapsin I (phospho S9), postsynaptic density-95 (PSD-95)) in brain tissue were detected. As shown in Fig. 4C–I, the Western blot results demonstrated that GABA sEV injection downregulated the protein expression of cleaved Caspase-3, Bax and Synapsin I (phospho S9) compared to that in the Ctrl sEV group. Conversely, the glutamate sEV injection upregulated the protein expression of cleaved Caspase-3, Bax and Synapsin I (phospho S9) compared to that in the Ctrl sEV group. The protein expression levels of Bcl-2, Synapsin I and PSD-95 were reversed. Thus, we concluded that GABA sEV treatment could alleviate Aβ toxicity, while glutamate sEV treatment could deteriorate Aβ toxicity.

### miR-132 in sEVs protected recipient neurons from Aβ toxicity

EVs represent an intercellular effector molecule exchange that allows transmitting cells to alter the expression of genes and proteins in receiving cells. Therefore, we compared the miRNA composition of sEVs isolated from glutamate/GABA/PBS-treated neurons through miRNA-sequencing.

**Fig. 5:**
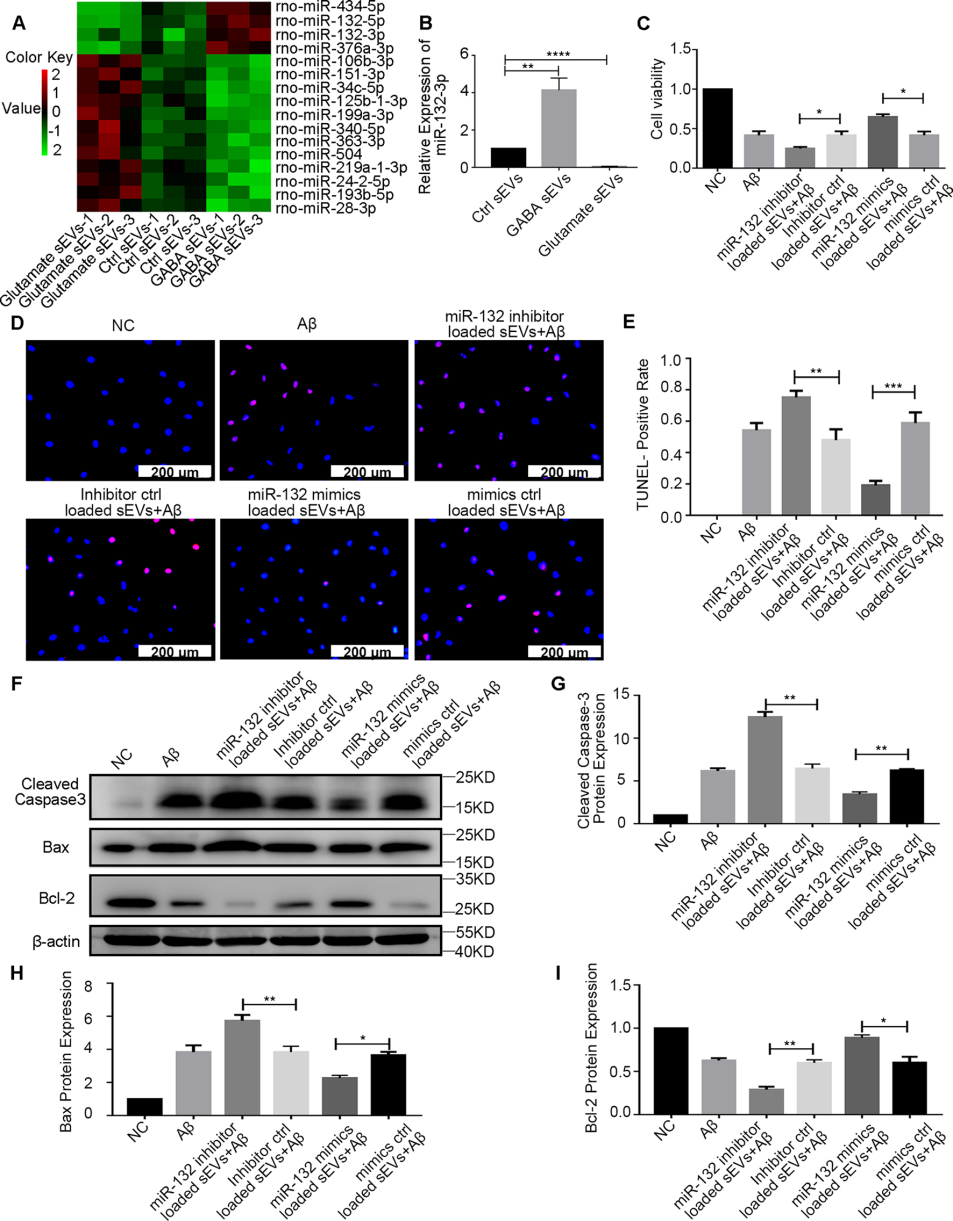
Identification of sEV miR-132 and its effect on Aβ pathology. **A** sEVs were isolated from glutamate (15 μM), GABA (500 μM), or PBS-treated neurons, and the miRNA composition of sEVs was compared by miRNA-sequencing. Heatmap of 16 significantly changed (GABA sEVs vs. Ctrl sEVs and Glutamate sEVs vs. Ctrl sEVs) miRNAs. **B** qPCR analysis of rno-miR-132-3p expression in sEVs. U6 was used as a reference gene. **C**–**E** Primary neurons were treated with miR-132 mimics, mimic control, miR-132 inhibitors, or inhibitor control-loaded sEVs for 24 h, followed by treatment with Aβ for 48 h. Cell viability was measured using CCK-8 (**C**) or TUNEL assays (**D**, **E**). **F**–**I** Expression of apoptotic molecules (cleaved Caspase-3, Bax, Bcl-2) in neurons treated with sEVs was detected by Western blot. n = 3. Data are presented as the mean ± SEM, *p < 0.05, **p < 0.01, ***p < 0.001, ****p < 0.0001

miRNA sequencing revealed 41 upregulated miRNAs in GABA sEVs compared to Ctrl sEVs and 14 downregulated miRNAs in glutamate sEVs compared to Ctrl sEVs. As shown in Additional file 5: Fig. S5A, 4 miRNAs overlapped in both groups. In addition, 64 downregulated miRNAs in GABA sEVs compared to Ctrl sEVs and 47 upregulated miRNAs in glutamate sEVs compared to Ctrl sEVs are shown in Additional file 5: Fig. S5B. Additionally, 12 miRNAs overlapped in both groups. Then, we combined the above 16 overlapping miRNAs (p < 0.05, Additional file 5: Fig. S5C) and constructed a heatmap (Fig. 5A). Next, qRT-PCR was performed to validate the expression of the most related miRNA, miR-132-3p. miR-132-3p is abundant in the brain, and accumulating evidence suggests that it plays a crucial role in synaptic plasticity, neurite outgrowth, and memory formation [21–23]. miR-132 deficiency occurs in AD and promotes its pathology [24, 25].

Pre-miRNA has two arms, 3p and 5p, respectively. In most cases, only one arm is processed as a mature miRNA. The miR-132-3p is the major miR-132 strand and is established as a neuroprotective miRNA. Conversely, miR132-5p is the minor (star, passive) strand expressed in neurons at much lower levels. Thus, miR-132-3p (in brief, mentioned as miR-132 in the following text) was selected for verification and to explore the underlying mechanism. qRT-PCR analysis (Fig. 5B) revealed a downregulation of miR-132 in the sEVs released by glutamate-treated neurons and an upregulation in the sEVs released by GABA-treated neurons compared to PBS-treated neurons.

To elucidate the role of miR-132 in sEVs in Aβ toxicity on neurons, we loaded the mimic negative control, miR-132 mimics, the inhibitor negative control, or miR-132 inhibitor into sEVs, followed by stimulation with Aβ. Next, CCK-8 and TUNEL assays were employed to detect the viability of the cultured neurons. The CCK-8 results showed that treatment with miR-132 mimic-loaded sEVs significantly upregulated neuronal viability compared to the mimic negative control. Conversely, the miR-132 inhibitor-loaded sEVs downregulated neuron viability compared to the inhibitor negative control (Fig. 5C). A similar pattern was also observed for the TUNEL assay (Fig. 5D, E). Additionally, the changes in apoptotic molecules caused by sEVs were detected (Fig. 5F–I). Treatment with miR-132 mimic-loaded sEVs significantly downregulated cleaved Caspase-3 and Bax expression in neurons compared to the mimic negative control. In contrast, miR-132 inhibitor-loaded sEVs upregulated cleaved Caspase-3 and Bax expression in neurons compared to the inhibitor negative control. The expression of Bcl-2 was reversed. These data supported the theory that miR-132 in sEVs exerted a protective effect against Aβ toxicity, while miR-132 deficiency in sEVs promoted Aβ toxicity.

**Fig. 6:**
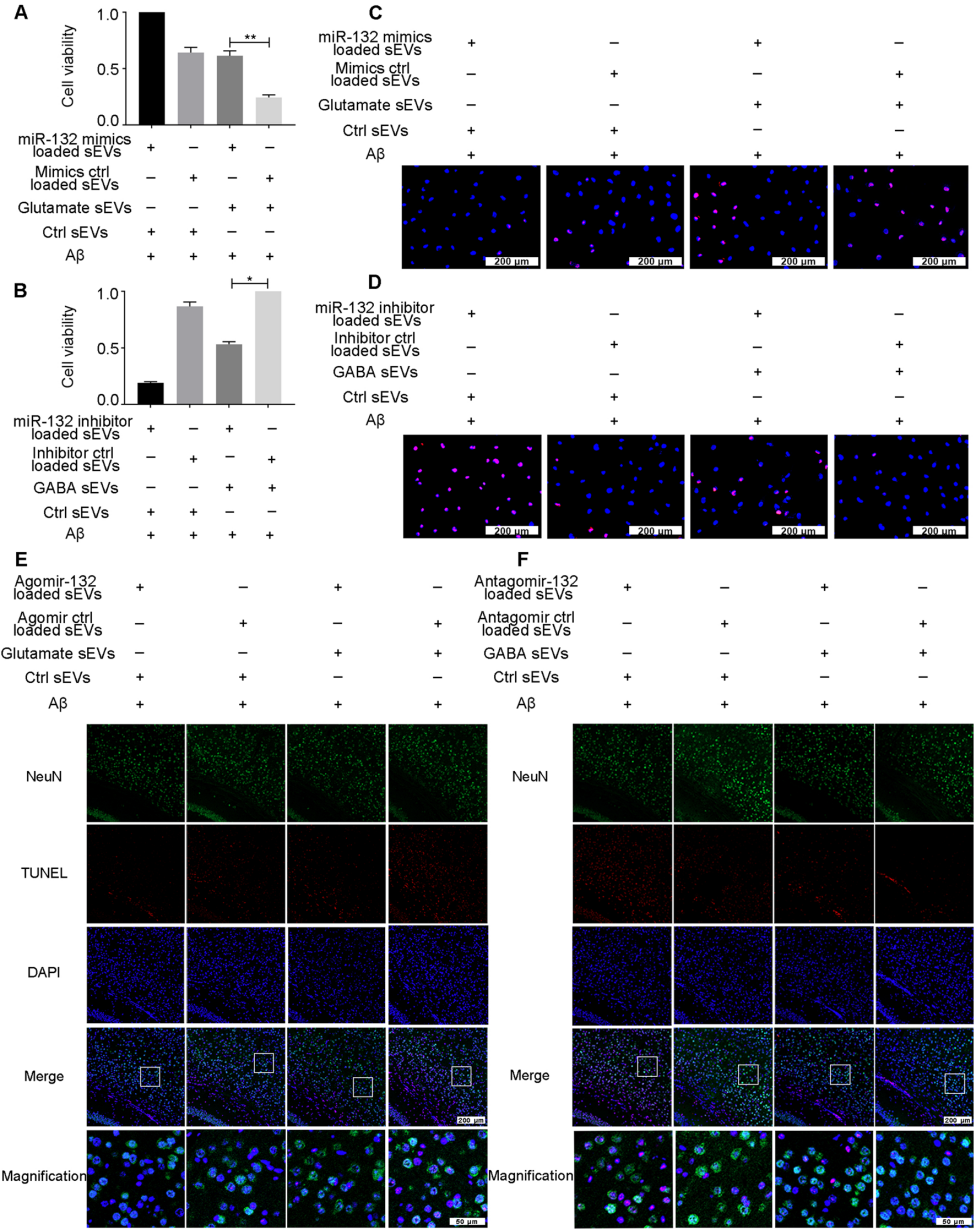
miR-132 deficiency in sEVs promotes Aβ pathology. **A**–**D** Under Aβ treatment, neurons were cotreated with glutamate sEVs and miR-132 mimic-loaded sEVs or GABA sEVs and miR-132 inhibitor-loaded sEVs. Then, cell viability was measured by CCK-8 (**A**, **B**) or TUNEL assays (**C**, **D**). **E**, **F** The mice were co-injected with glutamate sEVs/Ctrl sEVs and agomir-132/agomir ctrl loaded sEVs (**E**) and GABA sEVs/Ctrl sEVs and antagomir-132/antagomir ctrl (**F**)-loaded sEVs at every other day for seven consecutive intravenous injections. Then the proportion of TUNEL-positive cells in brain slices was calculated. n = 3. Data are presented as the mean ± SEM, *p < 0.05, **p < 0.01

Subsequently, we conducted rescue experiments, as shown in Fig. 6. Under Aβ treatment, when primary neurons were cotreated with glutamate sEVs and miR-132 mimic-loaded sEVs, but not mimic negative control-loaded sEVs, neuronal viability was significantly increased (Fig. 6A). Figure 6B shows that cotreatment with miR-132 inhibitor-loaded sEVs, but not inhibitor negative control-loaded sEVs, significantly diminished neuron viability. A similar pattern was observed in the TUNEL results (Fig. 6C, D; Additional file 6: Fig. S6A, B). Moreover, the changes in cleaved Caspase-3, Bax, and Bcl-2 caused by sEVs in neurons also supported the hypothesis that differential expression of miRNA-132 in sEVs might contribute to the occurrence of the phenomenon that the sEVs secreted by the GABA-treated neurons could alleviate Aβ-induced damage, while those released by the glutamate-treated neurons could aggravate the Aβ toxicity (Additional file 6: Fig. S6C–J).

Next, we further confirmed the rescue function of miR-132 mimic-loaded sEVs in glutamate sEVs on Aβ neurotoxicity in vivo. First, we confirmed that agomir-132 loaded sEVs could be effectively delivered to neurons in the brain. Referring to the methods described previously [26–28], Cy3-labeled agomir-132 was loaded into sEVs to track the fluorescence signal after injection into mice. Twenty-four hours after injection, the brains were harvested and the distribution of the Cy3 signal was measured (Additional file 7: Fig. S7A). The results indicated that Cy3-labeled agomir-132 loaded sEVs (red) and NeuN^+^-neurons (green) overlapped in the brain slices. We then gave the mice seven consecutive intravenous injections of the glutamate sEVs and miR-132 agomir-loaded sEVs or GABA sEVs and miR-132 antagomir-loaded sEVs loaded sEVs with the delivery system at intervals of 1 day apart. A similar pattern was observed in the in vivo results as in the in vitro rescue experiment (Fig. 6E, F, Additional file 6: Fig. S6K, L and Additional file 7: Fig. S7B–O).

These findings, taken together, showed that miR-132, which is rich in GABA-induced neuronal secretion of sEVs, has a protective effect, while the decreased level of miR-132 in the glutamate-induced neuronal secretion of sEVs could weaken the protective effect.

## Discussion

Based on previous studies, E/I balance is a critical issue in the nervous system. Intercommunication between E/I neurons forms the basis for functional neural networks, and the balance between overall excitation and inhibition within a network is central to normal cognition and memory. Any perturbation in the E/I ratio can cause abnormal network activity, which can lead to neurological symptoms [29]. The majority of excitatory signals are mediated by glutamate, which is the predominant neurotransmitter in the mammalian CNS. Glutamatergic neurotransmission is responsible for many cognitive, sensory and autonomic activities [30, 31]. Therefore, maintaining extracellular glutamate levels in a physiological range is crucial to ensure proper neuronal transmission and viability. Alterations in glutamate signaling transmission have been implicated in a large number of neurodegenerative diseases, including AD [32, 33]. In AD, E/I imbalance was initially thought to occur first, due to dysfunction of the glutamatergic and cholinergic systems. However, new evidence has demonstrated that the GABAergic system, the counterpart of E/I balance and the major inhibitory neurotransmitter system in the central nervous system, is altered significantly and that this contributes to E/I imbalance and further AD pathogenesis [1]. Lauterborn et al. assessed anatomical and electrophysiological synaptic E/I ratios in postmortem parietal cortex samples from middle-aged individuals with AD (early-onset), and revealed significantly elevated E/I ratios for AD, which supported the hypothesis that E/I imbalance contributes to the intellectual decline in AD [34]. Based on the above evidence, we believe that E/I balance is a critical issue. There is still much room for research on the underlying mechanism of how E/I imbalance causes AD pathology. Moreover, manipulating the GABAergic system to restore E/I balance and prevent neuronal excitotoxicity may also prevent the pathogenesis of AD or reduce the grade of dementia. Recent findings have indicated that communication mediated via sEVs is a common mechanism in the CNS [35], suggesting that manipulating the function of sEVs by different neurotransmitters regulates neuronal communication. We hypothesized that the function of sEVs might be regulated by the status of neurotransmitter balance and that sEVs might affect Aβ toxicity in neurons. Thus, in this study, we explored the effects of sEVs under E/I stimuli on the Aβ toxicity of recipient neurons.

It has been reported that cortical networks present immature types of synapses and low synaptic density at DIV 7. At DIV 14, the network became a mature type of synapse and exhibited a rich and stable burst pattern. Then, neurons connectivity decreased with age [36–38]. Moreover, glial cells gradually fell off as the culture time increased. Immunostaining cortical cultures at DIV 15 for the detection of GFAP^+^ positive glial cells (red) vs. NeuN^+^ neurons (green) revealed the staining of only 1–2% of cells, demonstrating minimal contamination by astrocytes (Additional file 1: Fig. S1B). This observation is in complete agreement with previous results [36, 39]. Therefore, we used cortical neurons cultured for 15 days to allow full differentiation and purification.

Specifically, under E/I stimuli, the neurons were continuously stimulated by either excitatory neurotransmitters or inhibitory neurotransmitters. This study found that the excitatory neurotransmitter glutamate and the inhibitory neurotransmitter GABA can stimulate neurons to release sEVs. However, the opposite effects of sEVs on Aβ toxicity were detected: sEVs secreted by GABA-stimulated neurons alleviate Aβ-induced damage, while sEVs derived from glutamate-stimulated neurons aggravate Aβ toxicity. Furthermore, the neuroprotection of sEVs secreted by neurons stimulated with different doses of GABA was similar, but the efficiency was different. As shown in the Additional file 3: Fig. S3 B, C, E, when the cells were treated with sEVs extracted from neurons treated with a low GABA concentration (100 μM), the sEVs exhibited a protective effect only when the total sEV protein concentration was high (80 μg/mL). However, as shown in the Additional file 3: Fig. S3 F, G, I, when the cells were treated with sEVs extracted from the neurons treated with a high GABA concentration (500 μM), the sEVs exerted a protective effect when the total sEVs protein concentration was low (20 μg/mL). The same was also found in sEVs secreted by glutamate-stimulated neurons (Additional file 3: Fig. 3B–D, F–H). Researchers have not studied the function of sEVs under the condition of E/I imbalance, but they have discussed the relationship between neurotransmitters and Aβ in detail. Notably, glutamate overstimulation is implicated in AD, and glutamate excitotoxicity causes neuronal cell death; numerous studies have shown that reducing the expression of glutamate in neurons can improve the cognitive function of AD model mice and reduce amyloid β (Aβ) plaques [32, 40–42]. It has also been demonstrated that GABAergic alterations play a critical role in AD pathogenesis; Ulrich et al. [43, 44] found that Aβ may weaken synaptic inhibition through the downregulation of GABA (A) receptors and that this effect is reversed in the presence of a GABA (A) receptor agonist. Our research provides new insights into the above mechanisms from the perspective of sEVs.

To exclude the factor of the copelleting of soluble proteins secreted by cells along with sEVs caused by ultracentrifugation, the incubation of neurons with conditioned medium depleted of sEVs by ultracentrifugation may be particularly appropriate as a negative control (NC). We compared the cell viability of the Aβ-treated neurons incubated with sEVs, sEV-depleted medium, and PBS (the same amount as the solvent of the sEVs). We found that in all three group sets (glutamate-sEVs, GABA-sEVs, Ctrl-sEVs), neurons incubated with sEV-depleted medium exhibited viability similar to that of neurons incubated with PBS (Additional file 1: Fig. S1D–F). This proved that the effect shown in the Additional file 1: Fig. S1E and F was truly sEV-related and not an artifact.

To confirm whether released sEVs can be internalized by other neurons, we labeled sEVs with the dyes CellMask and PKH26 [15]. The results of both dyes (Fig. 2A and Additional file 2: Fig. S2A) proved that the incubation of neurons with sEVs led to an uptake of sEVs. This was consistent with previous results of CellMask labeling sEVs [45].

As a type of EV, neuron-derived sEVs are membranous vesicles that can cross the BBB, can concentrate in the brain, and are generated within late endosomal compartments [9]. The release and absorption of sEVs are maintained in a dynamic balance. In the steady state, the entry of calcium ions through the synaptic receptor is a robust activator of multivesicular body (MVB) fusion to the plasma membrane, which promotes the secretion of extracellular bodies. After synapses are activated, the secretion of receptor-containing sEVs is enhanced, emphasizing that sEV release is a method of local elimination of receptors when synapses undergo plastic changes [46]. Therefore, when endosome fusion enhances synaptic receptors, continuous synaptic activation increases calcium ions in the dendritic axis. This, in turn, triggers the fusion of MVBs at the base of nearby synapses, producing cell receptors. The receptor pool disappears locally, thereby shrinking the synapse [15]. Interestingly, sEVs bring about intercellular effector molecule exchange, which allows transmitting cells to alter the expression of genes and proteins in receiving cells.

To monitor the functions of sEVs in which glutamate/GABA-stimulated neurons are secreted in a genetically-defined AD background, we injected APP/PS1 mice with sEVs and then evaluated the spatial learning and memory based on MWM performance. Subsequently, we concluded that GABA sEV treatment alleviates while glutamate sEV treatment deteriorates the spatial memory deficits in APP/PS1 mice based on the MWM test results. In addition, the biodistribution results of sEVs in vivo showed that sEVs were taken up in the tissues but also indicated that the brain is not the only target for exogenous sEVs. Thus, the effects on behavior might also be mediated by sEVs uptake and the modulation of additional non-CNS-related mechanisms.

Increasing evidence indicates that sEV-mediated intercellular communication plays a critical role in both physiological and pathological processes in neural systems. Therefore, we compared the miRNA composition of sEVs isolated from glutamate/GABA/PBS-treated neurons through miRNA sequencing. Combined with other results, the miRNA with a changing trend and the most significant difference, miR-132-3p, was selected for verification and mechanistic discussion. Furthermore, combined with the rescue experiment results, we speculated that although miR-132 in sEVs was not the sole functional substance in the multiple alterations, it plays a critical role in resisting Aβ toxicity. These data supported the theory that the effects of changes in Aβ toxicity on the neurons, spatial learning, and memory of mice were related to miR-132 in sEVs. Exploring that these changes in miR-132 in sEVs also participate in the molecular events leading to Aβ deposition would strengthen the evidence for a multiple-hit scenario for AD.

miRNAs function by combining with the 3’-untranslated region (3’-UTR) of mRNA targets. Therefore, a single miRNA could significantly modulate biological processes by targeting thousands of mRNA transcripts. Indeed, previous studies discovered various target genes of miR-132 in an AD model. miR-132 targets sirtuin and the deletion of miR-132 can foster Aβ production and plaque accumulation in a triple transgenic mouse AD model [47]. Furthermore, miR-132 inhibition induces apoptosis in cultured cortical and hippocampal primary neurons via PTEN/AKT/FOXO3 signaling [48]. Additionally, it has been demonstrated that the downregulation of miR-132 aggravates both amyloid and tau pathology in AD model mice and that it regulates the expression of inositol 1,4,5-trisphosphate 3-kinase B (ITPKB), a regulator of BACE1 activity [25]. miR-132 governs dendritic arborization, length, and spine density; impairs newborn neuron integration; and reduces synapse formation in the adult hippocampus by targeting the Rho family GTPase-activating protein p250GAP [49–51]. Here our study showed that miR-132 is at least one of the essential miRNAs in sEVs that were significantly effective in resisting Aβ toxicity. Therefore, the modulation of the levels of miR-132 by sEVs in patients might represent a promising, novel avenue for therapeutic intervention in AD.

## Conclusions

In summary, we proved that the function of sEVs could be regulated by the status of neurotransmitter balance and differentially affect Aβ toxicity in neurons. Our research provides new insights into the E/I imbalance that occurs in AD from the perspective of sEVs and suggests that engineering sEVs biologically by manipulating the GABAergic system may be a useful strategy to prevent or alleviate AD pathogenesis.

## Experimental procedures

### Primary neuronal isolation, culture, and reagents

Primary neuronal isolation was performed as described previously [52]. Typically, specific pathogen-free (SPF) E18 Sprague–Dawley (SD) rats aged 10–12 weeks were purchased from Shanghai Laboratory Animal Center (Shanghai, China). During the experiment, the pregnant rats were anesthetized by intraperitoneal injection of pentobarbital. The embryos were removed and placed individually in a Petri dish with cold Hank’s balanced salt solution (Beyotime, Shanghai, China). Then, the embryonic brains were dissected and immediately placed in a chilled container containing Dulbecco’s modified Eagle’s medium (DMEM, Gibco, Carlsbad, CA, USA). The cortices were isolated from the embryos under a dissecting microscope. Then, the tissues were digested using 0.125% trypsin for 30 min at 37 °C and agitated every 5 min; the digested tissues were finally placed in DMEM with 10% fetal bovine serum (FBS, Gibco). The cell suspension was then filtered through a 40-μm cell strainer, and the supernatant was collected by centrifugation at 1000 rpm for 5 min. The cell pellet was resuspended in DMEM containing 10% FBS. The cells were seeded into culture containers coated with poly-d-lysine (100 mg/mL; Sigma, Merck KGaA, Darmstadt, Germany) at a density of 6–7 × 10^5^ cells/mL and incubated in a humidified atmosphere at 37 °C and 5% CO_2_ for 4–6 h. The culture media was substituted with neurobasal medium (Gibco) supplemented with 1 × B27 (Gibco) and 1 × GlutaMAX (Gibco). Half of the media was replaced twice a week. The study protocol was approved by the Animal Care and Use Committee of The Tenth People’s Hospital of Shanghai (ID: SYXK 2011-0111) and Tongji University (Shanghai, China).

### Aβ treatment

Aβ_1–42_ was purchased in lyophilized form and resuspended at a concentration of 100 μM according to the manufacturer’s recommendation (China Peptides Co., Ltd, Shanghai, China). The soluble oligomers of Aβ_1–42_ were used as described previously [53]. We employed a CCK-8 assay, in which primary neurons were treated with different concentrations of Aβ_1–42_ oligomerization for 48 h to ascertain the toxic effect of Aβ on neurons. The results showed that the survival rate of primary neurons was significantly reduced post-Aβ treatment; the half-maximal inhibitory concentration (IC50) was approximately 0.5 μM. Unless indicated otherwise, the primary neurons were treated with a 0.5 μM concentration of the soluble oligomeric form of Aβ_1–42_ for 48 h. The IC50 was calculated using the online tool Quest Graph™ IC50 Calculator (https://www.aatbio.com/tools/ic50-calculator).

### Isolation of sEVs

sEVs were isolated from the supernatant of neurons as previously described [54] with some modifications. Briefly, the culture supernatants of the primary cortical neurons were collected. Then, the medium was centrifuged at 300×*g* for 10 min at room temperature to remove floating cells, 2000×*g* for 10 min at 4 °C to remove cell debris, and 10,000×*g* for 30 min 4 °C to remove large EVs and apoptotic bodies. The remaining supernatant was then ultracentrifuged at 100,000×*g* for 70 min at 4 °C in a 41Ti rotor (Beckman Coulter, Brea, CA) to obtain the sEV pellets. The final pellets were resuspended in 1 mL cold PBS and ultracentrifuged again. The sEV pellet was resuspended in 200 μL PBS for further analysis or experiments. The total protein amount of sEVs was measured by micro-bicinchoninic acid (BCA) colorimetric assays (Yeasen, stock number, 20201ES76; Shanghai, China) according to the manufacturer’s instructions.

### Nanoparticle tracking analysis (NTA)

The sEV concentration and size distribution were characterized by NTA with a ZetaView PMX 110 instrument (Particle Metrix, Germany) and its corresponding software (ZetaView 8.04.02 SP1), as published previously [55]. sEVs were prediluted in PBS to achieve a concentration within the 10^7^–10^8^ range for optimal analysis. The mean size and concentration (particles/mL) were recorded. For each sample, 500 μL of diluted EVs was extracted from the 10 mL supernatant secreted from 10^5^–10^6^ range neurons.

### sEVs labeling

The fluorescent dye DiR was purchased from Yeasen (stock number, 40757ES25; Shanghai, China), CellMask was purchased from Thermo Fisher (stock number, C10046, California, USA) and the PKH26 Red Fluorescent Kit was purchased from Sigma–Aldrich (stock number, MINI26). sEV labeling with DiR [56], PKH26 [57], or CellMask [45] was performed as described previously. Briefly, the purified sEVs were incubated in the presence of DiR (1 mM)/PKH26 (2 μM)/CellMask (5 mg/mL) for 15 min at 37 ℃, and the unbound dye was removed by ultracentrifugation at 120,000×*g* for 90 min. After PBS washes, the labeled sEVs were resuspended in PBS for subsequent use.

### Bicinchoninic acid (BCA)

After extracting protein from sEVs, cells or tissues, the protein concentration was measured using a BCA assay (Thermo Fisher Scientific) according to the manufacturer’s instructions.

### Western blot

Total proteins were extracted with lysis buffer (100 mM Tris–HCl, pH 6.8, 4% SDS, 20% glycerol). An equivalent of 5–30 μg protein/lane was separated by 10% sodium dodecyl sulfate–polyacrylamide gel electrophoresis (SDS-PAGE, Bio–Rad, Hercules, CA, USA) and transferred to polyvinylidene fluoride (PVDF) membranes (Thermo Fisher Scientific Inc., Waltham, Massachusetts, USA). PVDF membranes were blocked in 10% nonfat milk for 1 h, and probed with primary antibodies overnight at 4 °C. The membranes were washed with PBS containing 0.1% Tween 20 (PBST) three times and incubated with secondary antibodies for 1 h at room temperature. Then, the membranes were developed with enhanced chemiluminescence (ECL) detection reagents (Millipore, Billerica, MA, USA) and detected by Amersham Imager 600 (GE, Boston, USA). The primary antibodies were as follows: anti-CD63 monoclonal antibody (1:1000, ab217345, Abcam, Cambridge, UK), anti-Tsg101 monoclonal antibody (1:1000, Abcam, ab83), anti-GM130 antibody (1:200, sc-55591, Santa Cruz, USA), anti-TOMM20 antibody (1:1000, ab56783, Abcam), anti-COX IV antibody (1:1000, ab202554, Abcam), anti-β-actin (1:1000; 8H10D10, Cell Signaling Technology, Boston, USA), anti-cleaved Caspase-3 (1:1000, ab184787, Abcam), anti-Bax (1:1000, ab32503, Abcam), anti-Bcl-2 (1:1000, ab196495, Abcam), anti-Synapsin I (1:1000, ab254349, Abcam), anti-Synapsin I ((phospho S9) 1:1000, ab76260, Abcam), and anti-PSD-95 (1:1000, ab238135, Abcam). The secondary antibodies were anti-mouse IgG and anti-rabbit IgG (1:2000, Li-Cor Biosciences, Lincoln, USA). The intensity of the immunoreactive bands was quantified using ImageJ software (https://imagej.nih.gov/ij/, NIH, Bethesda, MD, USA).

### Cell viability assay

Cell viability was determined using the CCK-8 assay (Beyotime Biotechnology), according to the manufacturer’s instructions. Briefly, primary neurons were seeded into 96-well plates and treated at 37 °C. Then, the cells were incubated with CCK-8, and the absorbance was measured at 450 nm on a plate reader (Bio–Rad).

### TUNEL staining

Primary cortical neurons were collected and fixed in 4% paraformaldehyde (PFA) for 20 min, permeabilized with 0.1% Triton X-100, stained with DAPI (Sigma–Aldrich) at room temperature for 15 min, and subjected to TUNEL staining (Roche, Basel, Switzerland) at 37 °C for 1 h. Then, the cells were imaged under a fluorescence microscope. The apoptotic ratio was calculated as follows: apoptotic ratio = apoptotic neurons/total neurons × 100%.

### Reverse transcription qRT-PCR analysis

The expression of miRNAs was analyzed using quantitative reverse transcription PCR (qRT-PCR). Complementary DNA (cDNA) was synthesized using a PrimeScript™ RT reagent kit (TaKaRa RR037A, Dalian, China). Real-time PCR was performed using a real-time PCR kit (TaKaRa RR820A), followed by detection using a 7900HT fast RT-PCR instrument (Thermo Fisher Scientific), according to the manufacturer’s protocol. For the quantitative analysis of the expression of miRNAs, Bulge-Loop miRNA RT Primer was used. Bulge-Loop miRNA qRT-PCR primers were purchased from RiboBio (Guangzhou, China). The relative expression levels of miRNAs were normalized against that of U6. The expression levels were calculated using the 2^−∆∆CT^ method. The qRT-PCR primer sequences were as follows: miR-132-3p: 5′-TAACAGTCTACAGCCAT-3′ (forward) and 5′-CAGTGCGTGTCGTGGAGT-3′ (reverse); U6 5′-ACCACAGTCCATGCCATCAC-3′ (forward) and 5′-TCCACCACCCTGTTGC TGTA3′ (reverse).

### Animals and drug treatment

The experiments were performed on APP/PS1 male mice. APP/PS1 double transgenic mice were purchased from the Jackson Laboratory (Bar Harbor, Maine, USA, strain B6C3-Tg (APPswe, PS1dE9) 85Dbo/J; stock number, 004462). About 3–5 animals were housed per cage with free access to standard food and water and were maintained under standard laboratory conditions. The mice were allowed to adapt to the laboratory conditions before testing. The experiments were carried out in compliance with the Guidelines for Animal Care and Use in China, and the Animal Ethics Committee approved the protocols of Tongji University. Mice (20-weeks-old) were injected with glutamate sEVs or GABA sEVs through tail vein injection at the same time every other day. The dose of sEVs was 1.0 × 10^10^ particles/g body weight (p/g). The control mice were injected with Ctrl sEVs as a vehicle. Normal C57BL wild-type mice and AD model groups treated with PBS were included as negative and positive controls.

### Live animal imaging

APP/PS1 male mice were used. Freshly purified DiR-labeled sEVs were injected intravenously (i.v.) through the tail vein at the dose of 1.0 × 10^10^ p/g. For the analysis of DiR-sEV distribution, an AniView100 Multimode Live Animal Imaging System (Boluteng Biological Technology Co., Ltd, Guangzhou, China) was used. At 24 h postinjection, the mice were anesthetized with pentobarbital, and fluorescence images were captured. For the perfusion experiment, the mice were sedated, and the vascular system was flushed by transcardial perfusion. The left ventricle was infused with PBS (5 mL/min), and the right atrium was perforated. The outflow liquid, liver, and tail were monitored during the procedure to ensure successful perfusion. After 5 min of perfusion, the organs were harvested and imaged. The live mice or the harvested organs were imaged for 12 s (excitation 710 nm, emission 760 nm). The data were analyzed with the corresponding software AniView100 (Guangzhou, China).

### MWM test

Spatial learning and memory were evaluated using the MWM test 40 days post sEV administration. Briefly, a black circular pool (150 cm in diameter and 50 cm in depth), partially filled with opaque water and set at 26 ± 1 °C, was divided equally into 4 quadrants for the test. An escape platform (8 cm in diameter) was placed 1.5 cm below the surface of the water in the middle of one of the quadrants (goal quadrant). A digital camera connected to a video recorder and tracking device was suspended above the center of the pool to track the swimming trajectories and transfer the parameters to electronic imaging analysis software. A total of 12 mice were tested in each group.

The animals underwent four consecutive trials each day over a 5-day memory acquisition trial (training). Then, the mice were trained to swim four times/day to find the hidden platform. In every trial, each mouse was released at one starting position and faced the wall of the maze. From the four different quadrants, we recorded the escape latency of the mouse, which was the time that the mouse spent on finding out and reaching the platform. If the mouse failed to find the platform in standard time (60 s), the observer would help the animal reach the platform; then, the mouse continued to the next experiment after resting on the platform for 20 s. In this case, the latency was recorded as 60 s. This procedure was repeated for all four start locations. On day 6, the mice were tested on a 90-s spatial probe trial, in which the platform was removed. All the mice were placed in a quadrant opposite to the target quadrant and allowed to swim freely. The time spent in the target quadrant and the number of times the mice crossed the platform position were recorded.

### Immunofluorescence

Mice were fixed by transcardial perfusion with 4% formaldehyde, and the brains were cut into 30-µm slices and placed on slides. The cells were cultured on coverslips. Briefly, the slides or coverslips were washed with PBS and fixed for 15 min in 4% formaldehyde at room temperature. After washing with PBS, the cells were permeabilized with 0.1% Triton X-100 in PBS for 5 min and washed again with PBS. Then, the cells were blocked in 0.5% bovine serum albumin (BSA) in PBS for 30 min prior to staining with primary antibodies overnight at 4 °C. After washing with PBS, the cells were incubated with secondary antibody in PBS for 1 h. Nuclei were stained with NeuN (1:200, ab177487, Abcam), and neuronal frame structures were stained with class III β-tubulins (1:200, β III -tubulin, ab18207, Abcam) and GFAP (1:200, ab4674, Abcam). Images were captured under a DM6000 fluorescence microscope (Leica, Wetzlar, USA).

### sEV loading

After 14 days of culture in vitro, the primary neurons became mature, and the sEVs were collected. The miRNAs were loaded into sEVs using SBI’s high-efficiency sEVs-Fect™ siRNA/miRNA Transfection Kit (stock number: EXFT200A-1; Palo Alto, USA). The workflow consisted of two steps according to the manufacturer’s instructions. Step 1: Load miR-132 mimics or inhibitors into already isolated sEVs. The miR-132 mimics or inhibitors were incubated with sEVs-Fect siRNA/miRNA Transfection reagent for 15 min at room temperature. Then, the isolated sEVs were added to the mixture and incubated at 37 °C for 1 h. sEVs at a total protein concentration of 1 mg (measured by BCA) were mixed with 300 μg of miR-132 mimic or 700 μg inhibitor in transfection reagent. Step 2: Clean up the reaction by removing free mimics or inhibitors, transfection reagent, and free siRNA/miRNA-sEVs-Fect complexes. The reaction was transferred to the prewashed spin column (Beckman Coulter, USA) and incubated with gentle rotation for 10 min at room temperature. Subsequently, the sEVs were collected by the centrifugation of the spin column for 30 s at 1000×*g*. The loaded EVs were then ready for use in downstream applications.

Similarly, agomir-132 and antagomir-132 were loaded into sEVs and were intravenously injected into APP/PS1 mice. A total of 300 μg of agomir-132 or 700 μg of antagomir-132 were encapsulated in 1 mg of sEVs. We then administered seven consecutive intravenous injections of agomir-132- or antagomir-132-loaded sEVs to the mice at intervals of 1 day apart.

Cy3-labeled agomir-132 (red) was injected into APP/PS1 mice. The brain was harvested after 24 h to confirm whether agomir-132 loaded sEVs could be effectively delivered to neurons in brain.

The miR-132 mimic and its control; miR-132 inhibitor and its control; agomir-132 and its control; antagomir-132 inhibitor and its control; and Cy3-labeled agomir-132 were designed and synthesized by RiboBio (Shanghai, China).

### Small RNA sequencing

The experimental procedure was performed in accordance with the standard steps provided by Illumina, including library preparation and sequencing. The small RNA sequencing library was prepared using TruSeq Small RNA Sample Prep Kits (Illumina, San Diego, CA, USA). The constructed library was sequenced using an Illumina Hiseq 2000/2500, and the sequencing read length was 50 bp single-ended. The miRNA data analysis software was ACGT101-miR provided by Lianchuan Biological Company (Hangzhou, China). MiRNA differential analysis was carried out, and a Venn diagram and a heatmap were constructed.

### Statistical analysis

Data were plotted, analyzed using GraphPad Prism 8 software (GraphPad), and presented as mean ± standard error of the mean (SEM). Statistical significance was determined by unpaired Student’s t-test for two groups or one-way analysis of variance (ANOVA) for multiple groups. Differences were considered statistically significant when the *p*-value was < 0.05. All experiments were repeated at least three times.

## Supplementary Information

**Additional file 1: Fig. S1.** sEVs-depleted medium had no effect on the cell viability of Aβ-treated neurons. (A) Image of cultured primary cortical neurons on DIV 15. (B) Immunostaining of cortical cultures on DIV 15 for the detection of GFAP^+^ glial cells (red) vs. NeuN^+^ neurons (green). (C) The morphology of sEVs from glutamate or GABA-treated neurons was observed under a transmission electron microscope. Glutamate sEVs = sEVs derived from 10 μM glutamate-treated neurons, GABA sEVs = sEVs derived from 300 μM GABA-treated neurons. (D-F) Neurons were incubated with PBS (the same amount as the solvent of the sEVs), sEVs and sEV-depleted medium (Negative control, NC), then with Aβ as before. Neuronal viability in Ctrl-sEV-treated group (D), glutamate-sEV-treated group (E), GABA-sEV-treated group (F) and NC group were compared using CCK-8. n = 3. Data are presented as the mean ± SEM, ***p < 0.001, ****p < 0.0001.

**Additional file 2: Fig. S2.** Internalization of PKH26 labeled sEVs in primary cultured neurons. (A) sEVs were labeled with PKH26 (red) and added to primary neurons. A control group (only PKH26 without sEVs) was established to exclude false-positive staining caused by the dye. Then, the neurons were immunostained with βIII-tubulin (green) and visualized under a confocal microscope. PKH26 -labeled sEVs were indicated by white arrows. n = 3.

**Additional file 3: Fig. S3.** Function of sEVs from neurons treated by various concentrations of glutamate or GABA. (A) Primary neurons on DIV 15 were treated with a gradient concentration of Aβ_1–42_ for 48 h. Cell viability was measured using a CCK-8 assay. (B–I) sEVs were isolated from low concentrations of glutamate (5 μM)/GABA (100 μM), or high concentrations of glutamate (15 μM)/GABA (500 μM) and added to the culture media. Cell viability was measured using CCK-8 (D, E, H, I) and TUNEL assays (B, C, F, G). Glutamate5 sEVs = sEVs derived from 5 μM glutamate treated neurons, GABA100 sEVs = sEVs derived from 100 μM GABA-treated neurons, Glutamate15 sEVs = sEVs derived from 15 μM glutamate-treated neurons, GABA500 sEVs = sEVs derived from 500 μM GABA treated neurons, Ctrl sEVs = sEVs derived from PBS-treated neurons. n = 3. Data are presented as the mean ± SEM, *p < 0.05, **p < 0.01, ***p < 0.001.

**Additional file 4: Fig. S4.** Internalization of PKH-26 labeled sEVs by neurons in APP/PS1 mice. (A) sEVs were labeled with PKH26 (red) and injected via the tail vein in mice. Brain slices were harvested and subjected to the immunostaining of the neuron marker NeuN (green) and visualized. The control group mice were injected with PKH26 only without sEVs. n = 3.

**Additional file 5:** **Fig. S5.** sEV miRNA expression profile. (A–C) sEVs were isolated from glutamate (15 μM), GABA (500 μM), or PBS-treated neurons, and the miRNA composition of sEVs was compared by miRNA-sequencing. (A) miRNA sequencing showed 41 upregulated miRNAs in GABA sEVs and 14 downregulated miRNAs in glutamate sEVs compared to Ctrl sEVs. 4 repeated miRNAs were identified in both groups. (B) There were 64 downregulated miRNAs in GABA sEVs and 47 upregulated miRNAs in glutamate sEVs compared with Ctrl sEVs. A total of 12 miRNAs overlapped in both groups. (C) An aggregate of 16 miRNAs (p < 0.05).

**Additional file 6: Fig. S6.** sEVs with inhibited or over-expressed miR-132 abrogated Glutamate/GABA sEVs induced alterations of apoptotic molecules in Aβ treated neurons, respectively. (A, B) TUNEL- positive rate of neurons in Fig. 6C and D were calculated. (C–J) Neurons were cotreated with glutamate sEVs and miR-132 mimic-loaded sEVs (C, E–G) or GABA sEVs and miR-132 inhibitor-loaded sEVs (D, H–J). 24 h later, neurons were subjected to Aβ treatment for another 48 h. Then, the expression of apoptotic molecules (cleaved Caspase-3, Bax, and Bcl-2). (K, L) The proportion of TUNEL-positive cells in brain slices in Fig. 6E and F was detected. n = 3. Data are presented as the mean ± SEM, *p < 0.05, **p < 0.01, ***p < 0.001.

**Additional file 7: Fig. S7.** sEVs with inhibited or over-expressed miR-132 abrogated Glutamate/GABA sEVs induced alterations of apoptotic molecules and synapse-related proteins in APP/PS1 mice, respectively. (A) Cy3-labeled agomir-132 was loaded into sEVs and injected into mice. Twenty-four hours later, the brain slices showed overlapped Cy3-labeled agomir-132-loaded sEVs (red) and NeuN+-neurons (green). (B-O) The mice were injected with glutamate sEVs/Ctrl sEVs and agomir-132/agomir ctrl (B-H) and GABA sEVs/Ctrl sEVs and antagomir-132/antagomir ctrl (I-O)-loaded sEVs at every other day for seven consecutive injections. The expression of apoptotic molecules (cleaved Caspase-3, Bax, and Bcl-2) and synapse-related proteins (Synapsin I, Synapsin I (phospho S9), postsynaptic density-95 (PSD-95)) in the brain lysate of mice was examined by Western blot. n = 3. Data are presented as the mean ± SEM, *p < 0.05, **p < 0.01, ***p < 0.001.

## Data Availability

The data that support the findings of this study are available from the corresponding author upon reasonable request.

## Abbreviations

AD: Alzheimer’s disease
Aβ: Amyloid β-protein
EVs: Extracellular vesicles
sEVs: Small extracellular vesicles
GABA: Gamma-aminobutyric acid
APP: Amyloid precursor protein
BACE1: β-site APP-cleaving enzyme 1
BBB: Blood–brain barrier
CNS: Central nervous system
miRNAs: MicroRNAs
DIV: Day in vitro
MVB: Multivesicular bodies
ITPKB: Inositol 1,4,5-trisphosphate 3-kinase B
NTA: Nanoparticle tracking analysis
TUNEL: TdT-mediated dUTP Nick-End Labeling
CCK-8: Cell counting kit-8
IC50: Half-maximal inhibitory concentration
DiR: 1, 1′-Dioctadecyl-3, 3, 3′, 3′-tetramethylindotricarbocyanine iodide
ELISA: Enzyme-linked immunosorbent assay
PSD-95: Postsynaptic density-95
qRT-PCR: Quantitative real-time polymerase chain reaction
3’-UTR: 3’-Untranslated region
SPF: Specific pathogen-free
SD: Sprague–Dawley
