# Supplementary figures and images for: Neurotransmitter-stimulated neuron-derived sEVs have opposite effects on amyloid β-induced neuronal damage
Source: J Nanobiotechnology. 2021 Oct 15;19:324. doi: 10.1186/s12951-021-01070-5 (PMC8518222; doi:10.1186/s12951-021-01070-5)

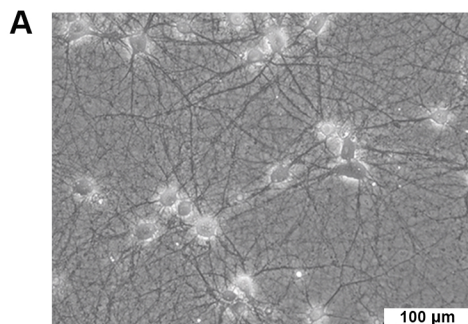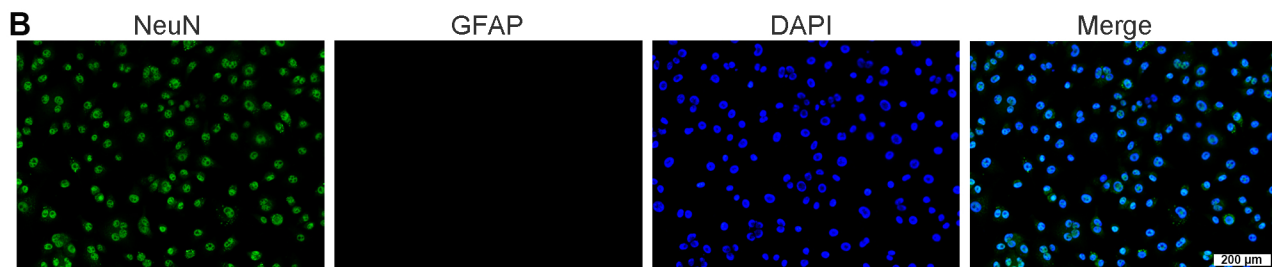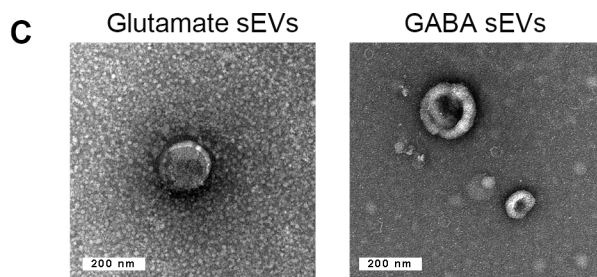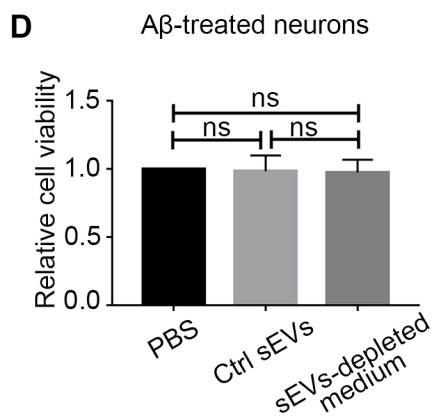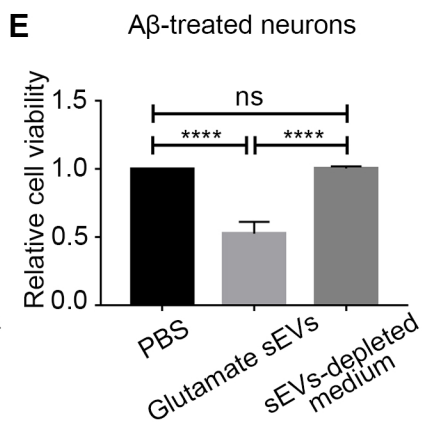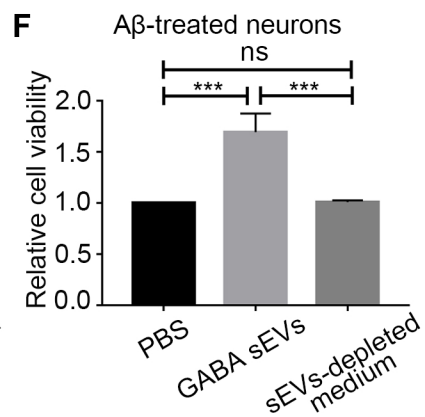

Supplement: Supplementary file 1 — Additional file 1: Fig. S1. sEVs-depleted medium had no effect on the cell viability of Aβ-treated neurons. (A) Image of cultured primary cortical neurons on DIV 15. (B) Immunostaining of cortical cultures on DIV 15 for the detection of GFAP+ glial cells (red) vs. NeuN+ neurons (green). (C) The morphology of sEVs from glutamate or GABA-treated neurons was observed under a transmission electron microscope. Glutamate sEVs = sEVs derived from 10 μM glutamate-treated neurons, GABA sEVs = sEVs derived from 300 μM GABA-treated neurons. (D-F) Neurons were incubated with PBS (the same amount as the solvent of the sEVs), sEVs and sEV-depleted medium (Negative control, NC), then with Aβ as before. Neuronal viability in Ctrl-sEV-treated group (D), glutamate-sEV-treated group (E), GABA-sEV-treated group (F) and NC group were compared using CCK-8. n = 3. Data are presented as the mean ± SEM, ***p < 0.001, ****p < 0.0001. [file 12951_2021_1070_MOESM1_ESM.pdf]

**A**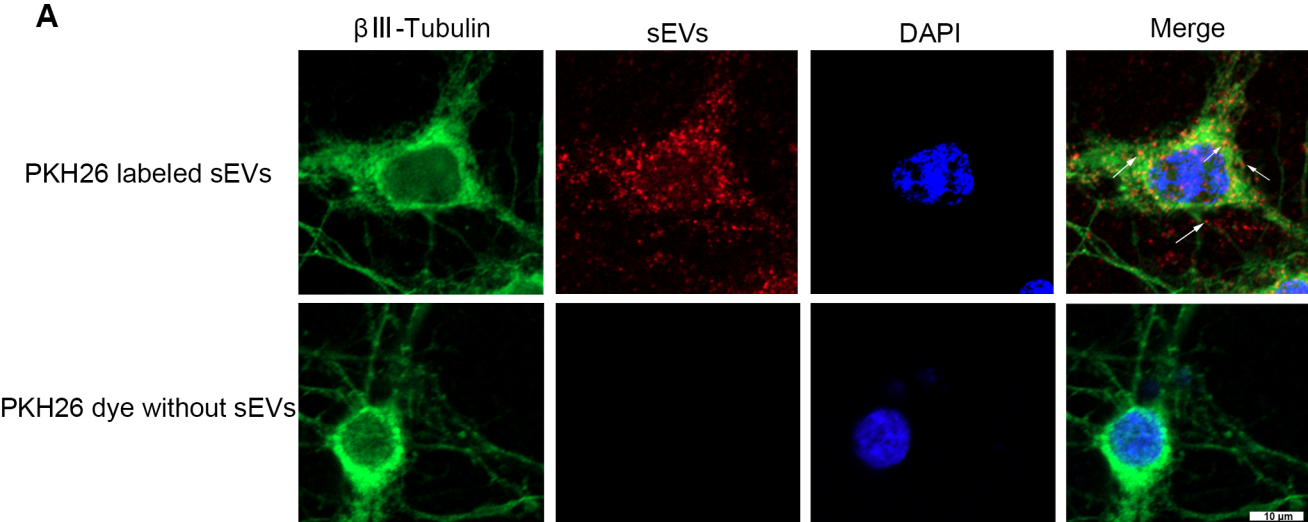

Supplement: Supplementary file 2 — Additional file 2: Fig. S2. Internalization of PKH26 labeled sEVs in primary cultured neurons. (A) sEVs were labeled with PKH26 (red) and added to primary neurons. A control group (only PKH26 without sEVs) was established to exclude false-positive staining caused by the dye. Then, the neurons were immunostained with βIII-tubulin (green) and visualized under a confocal microscope. PKH26 -labeled sEVs were indicated by white arrows. n = 3. [file 12951_2021_1070_MOESM2_ESM.pdf]

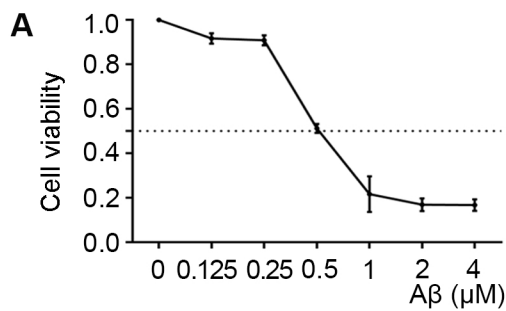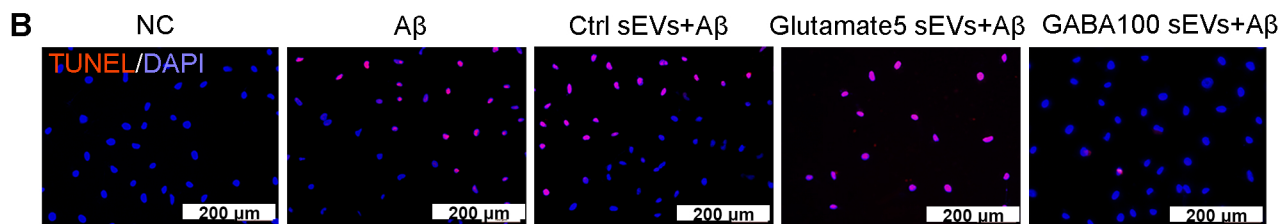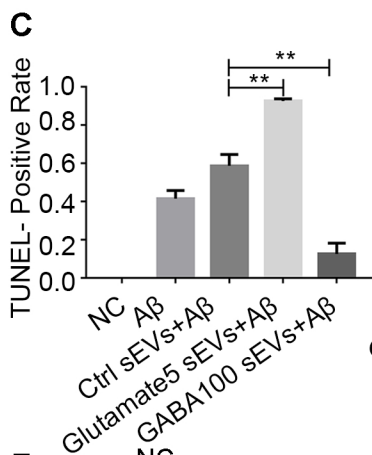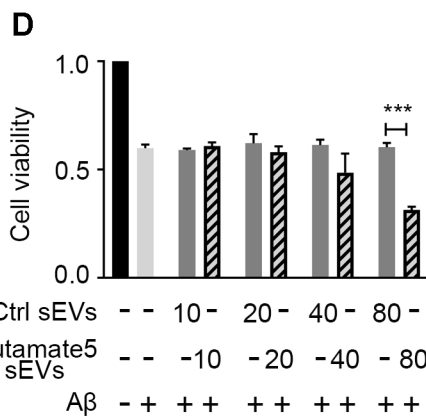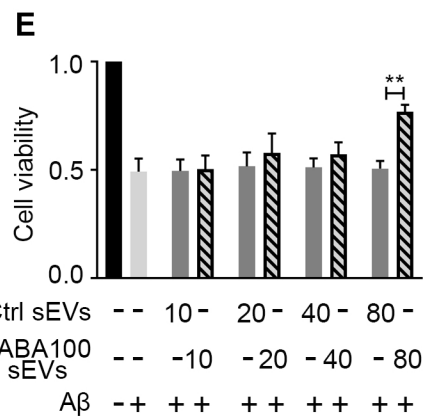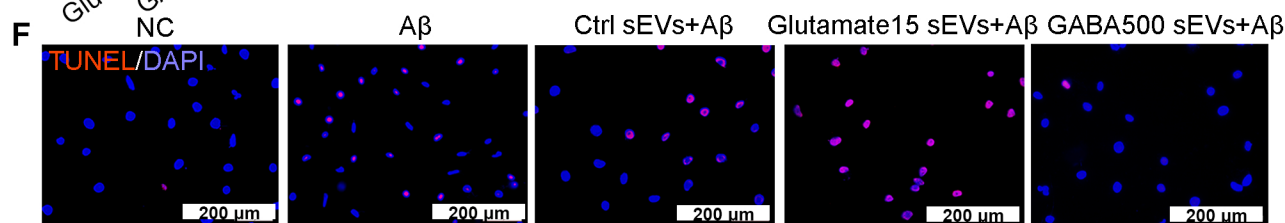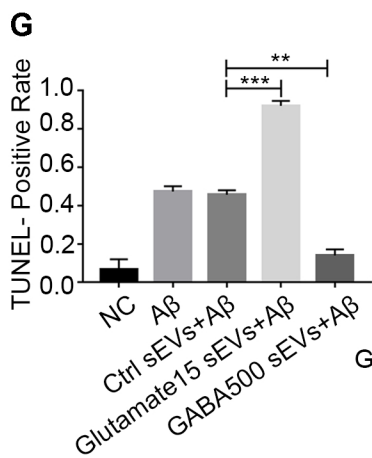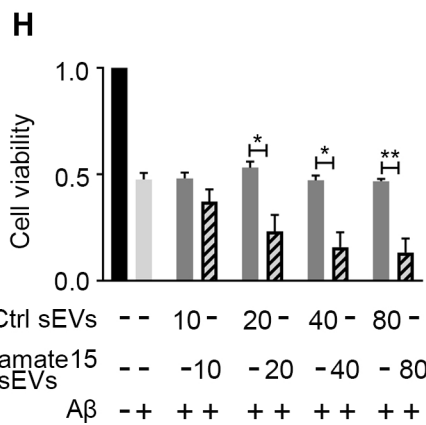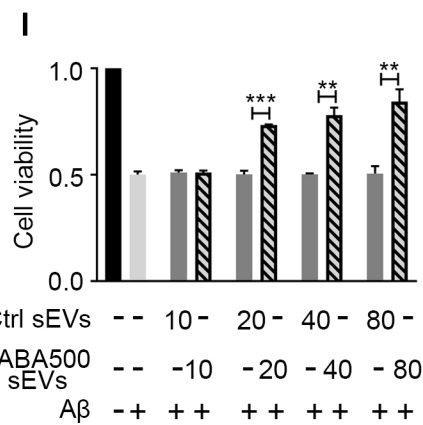

Supplement: Supplementary file 3 — Additional file 3: Fig. S3. Function of sEVs from neurons treated by various concentrations of glutamate or GABA. (A) Primary neurons on DIV 15 were treated with a gradient concentration of Aβ1–42 for 48 h. Cell viability was measured using a CCK-8 assay. (B–I) sEVs were isolated from low concentrations of glutamate (5 μM)/GABA (100 μM), or high concentrations of glutamate (15 μM)/GABA (500 μM) and added to the culture media. Cell viability was measured using CCK-8 (D, E, H, I) and TUNEL assays (B, C, F, G). Glutamate5 sEVs = sEVs derived from 5 μM glutamate treated neurons, GABA100 sEVs = sEVs derived from 100 μM GABA-treated neurons, Glutamate15 sEVs = sEVs derived from 15 μM glutamate-treated neurons, GABA500 sEVs = sEVs derived from 500 μM GABA treated neurons, Ctrl sEVs = sEVs derived from PBS-treated neurons. n = 3. Data are presented as the mean ± SEM, *p < 0.05, **p < 0.01, ***p < 0.001. [file 12951_2021_1070_MOESM3_ESM.pdf]

**A**

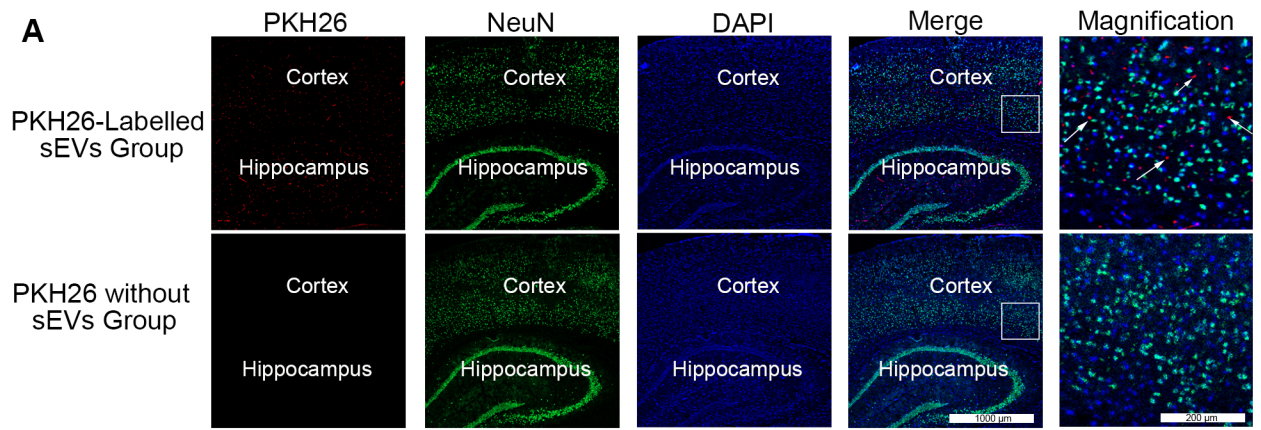

Supplement: Supplementary file 4 — Additional file 4: Fig. S4. Internalization of PKH-26 labeled sEVs by neurons in APP/PS1 mice. (A) sEVs were labeled with PKH26 (red) and injected via the tail vein in mice. Brain slices were harvested and subjected to the immunostaining of the neuron marker NeuN (green) and visualized. The control group mice were injected with PKH26 only without sEVs. n = 3. [file 12951_2021_1070_MOESM4_ESM.pdf]

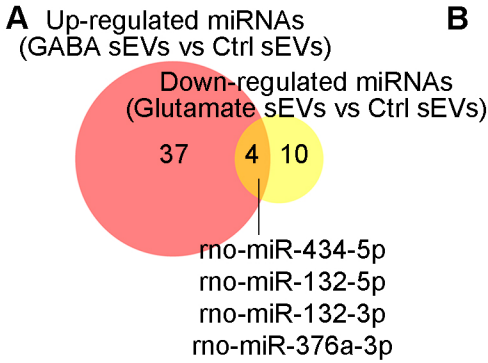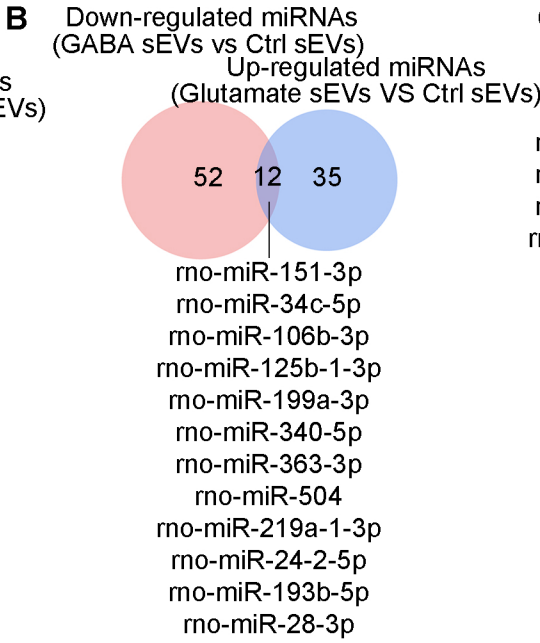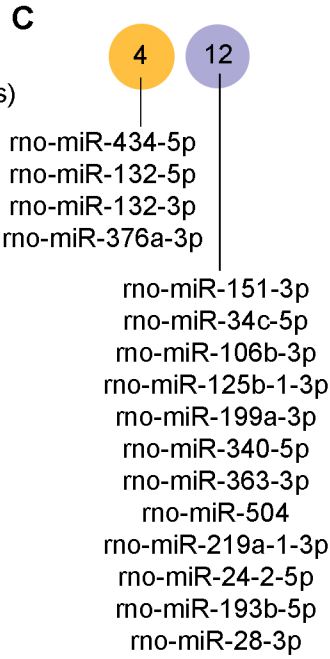

Supplement: Supplementary file 5 — Additional file 5: Fig. S5. sEV miRNA expression profile. (A–C) sEVs were isolated from glutamate (15 μM), GABA (500 μM), or PBS-treated neurons, and the miRNA composition of sEVs was compared by miRNA-sequencing. (A) miRNA sequencing showed 41 upregulated miRNAs in GABA sEVs and 14 downregulated miRNAs in glutamate sEVs compared to Ctrl sEVs. 4 repeated miRNAs were identified in both groups. (B) There were 64 downregulated miRNAs in GABA sEVs and 47 upregulated miRNAs in glutamate sEVs compared with Ctrl sEVs. A total of 12 miRNAs overlapped in both groups. (C) An aggregate of 16 miRNAs (p < 0.05). [file 12951_2021_1070_MOESM5_ESM.pdf]

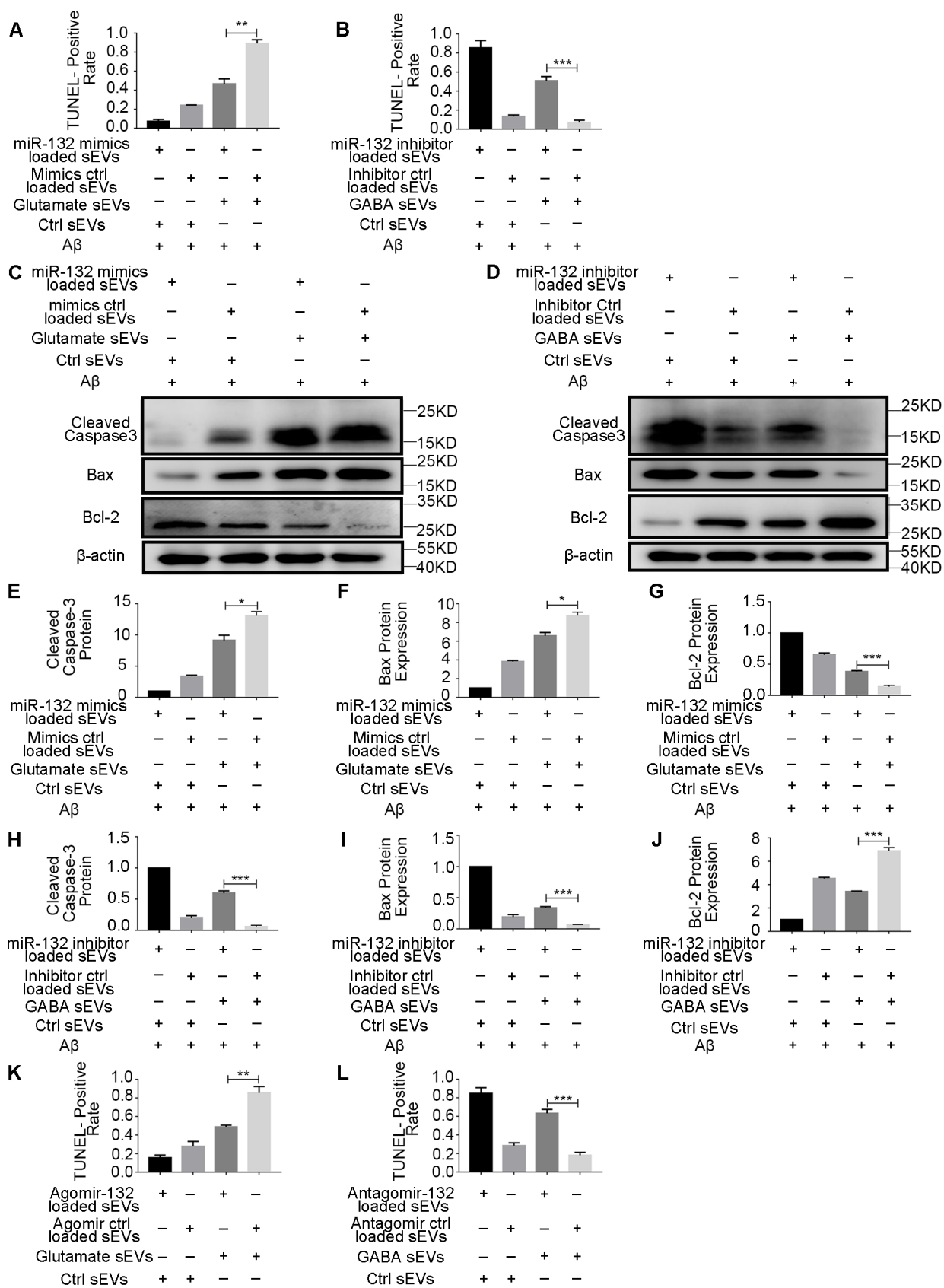

Supplement: Supplementary file 6 — Additional file 6: Fig. S6. sEVs with inhibited or over-expressed miR-132 abrogated Glutamate/GABA sEVs induced alterations of apoptotic molecules in Aβ treated neurons, respectively. (A, B) TUNEL- positive rate of neurons in Fig. 6C and D were calculated. (C–J) Neurons were cotreated with glutamate sEVs and miR-132 mimic-loaded sEVs (C, E–G) or GABA sEVs and miR-132 inhibitor-loaded sEVs (D, H–J). 24 h later, neurons were subjected to Aβ treatment for another 48 h. Then, the expression of apoptotic molecules (cleaved Caspase-3, Bax, and Bcl-2). (K, L) The proportion of TUNEL-positive cells in brain slices in Fig. 6E and F was detected. n = 3. Data are presented as the mean ± SEM, *p < 0.05, **p < 0.01, ***p < 0.001. [file 12951_2021_1070_MOESM6_ESM.pdf]

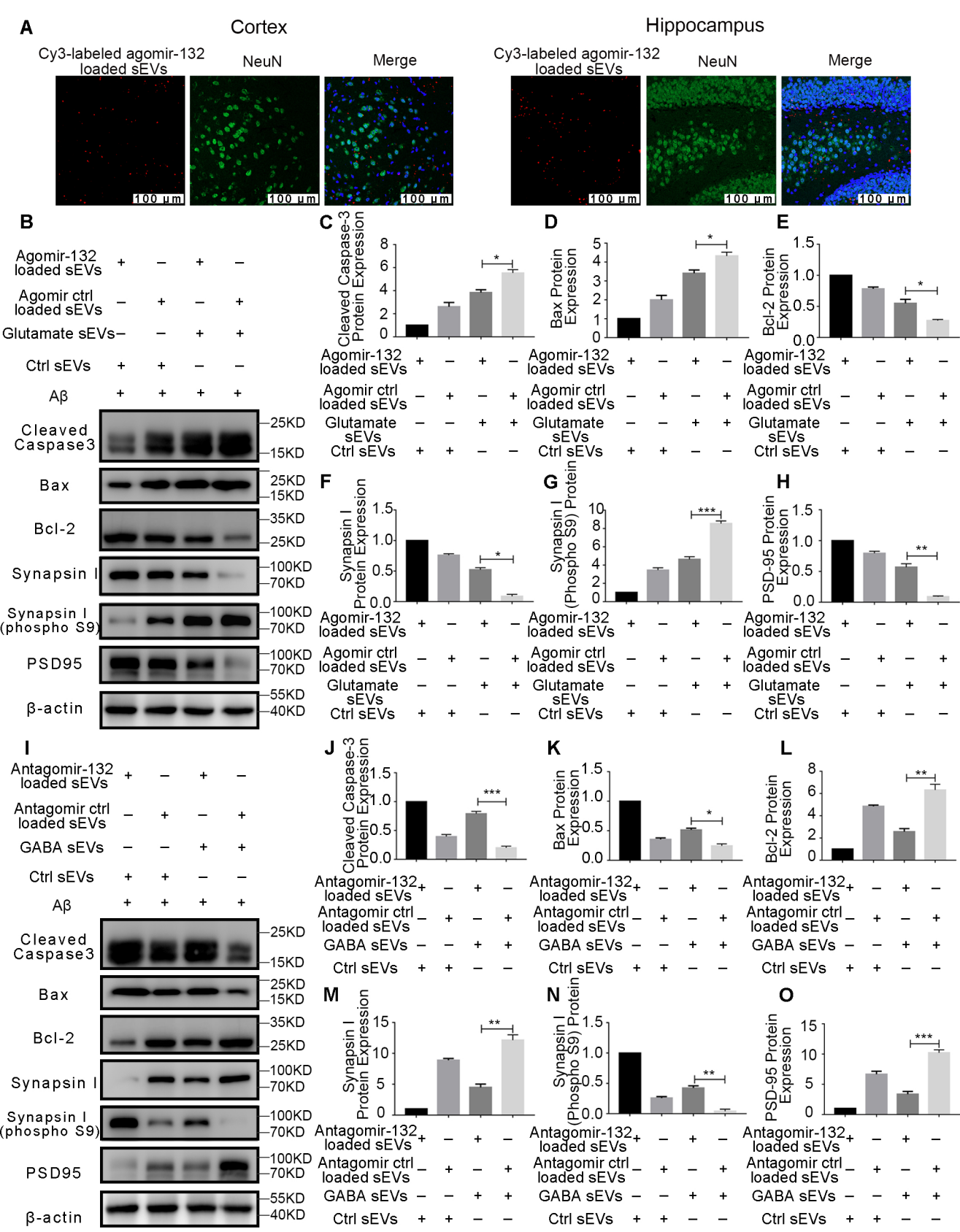

Supplement: Supplementary file 7 — Additional file 7: Fig. S7. sEVs with inhibited or over-expressed miR-132 abrogated Glutamate/GABA sEVs induced alterations of apoptotic molecules and synapse-related proteins in APP/PS1 mice, respectively. (A) Cy3-labeled agomir-132 was loaded into sEVs and injected into mice. Twenty-four hours later, the brain slices showed overlapped Cy3-labeled agomir-132-loaded sEVs (red) and NeuN+-neurons (green). (B-O) The mice were injected with glutamate sEVs/Ctrl sEVs and agomir-132/agomir ctrl (B-H) and GABA sEVs/Ctrl sEVs and antagomir-132/antagomir ctrl (I-O)-loaded sEVs at every other day for seven consecutive injections. The expression of apoptotic molecules (cleaved Caspase-3, Bax, and Bcl-2) and synapse-related proteins (Synapsin I, Synapsin I (phospho S9), postsynaptic density-95 (PSD-95)) in the brain lysate of mice was examined by Western blot. n = 3. Data are presented as the mean ± SEM, *p < 0.05, **p < 0.01, ***p < 0.001. [file 12951_2021_1070_MOESM7_ESM.pdf]
